# Modulating the Luminescence, Photosensitizing Properties, and Mitochondria-Targeting Ability of D-π-A-Structured Dihydrodibenzo[*a*,*c*]phenazines

**DOI:** 10.3390/molecules28176392

**Published:** 2023-09-01

**Authors:** Zhaozhi Zhang, Qijing Wang, Xinyi Zhang, Dong Mei, Ju Mei

**Affiliations:** 1 Key Laboratory for Advanced Materials, Feringa Nobel Prize Scientist Joint Research Center, Frontiers Science Center for Materiobiology and Dynamic Chemistry, Joint International Research Laboratory for Precision Chemistry and Molecular Engineering, Institute of Fine Chemicals, School of Chemistry and Molecular Engineering, East China University of Science & Technology, 130 Meilong Road, Shanghai 200237, China; zzzhang97@163.com (Z.Z.); y30210358@mail.ecust.edu.cn (Q.W.); chang_hsini@163.com (X.Z.); 2 Clinical Research Center, Beijing Children’s Hospital, Capital Medical University, National Center for Children’s Health, Beijing 100045, China

**Keywords:** aggregation-induced emission, vibration-induced emission, photodynamic therapy, mitochondria-specific imaging, photosensitizing properties, dihydrodibenzo[*a*,*c*]phenazines

## Abstract

Herein, pyridinium and 4-vinylpyridinium groups are introduced into the VIE-active *N*,*N*′-disubstituted-dihydrodibenzo[*a*,*c*]phenazines (DPAC) framework to afford a series of D-π-A-structured dihydrodibenzo[*a*,*c*]phenazines in consideration of the aggregation-benefited performance of the DPAC module and the potential mitochondria-targeting capability of the resultant pyridinium-decorated DPACs (DPAC-PyPF_6_ and DPAC-D-PyPF_6_). To modulate the properties and elucidate the structure–property relationship, the corresponding pyridinyl/4-vinylpyridinyl-substituted DPACs, i.e., DPAC-Py and DPAC-D-Py, are designed and studied as controls. It is found that the strong intramolecular charge transfer (ICT) effect enables the effective separation of the highest occupied molecular orbital (HOMO) and the lowest unoccupied molecular orbital (LUMO) of DPAC-PyPF_6_ and DPAC-D-PyPF_6_, which is conducive to the generation of ROS. By adjusting the electron-accepting group and the π-bridge, the excitation, absorption, luminescence, photosensitizing properties as well as the mitochondria-targeting ability can be finely tuned. Both DPAC-PyPF_6_ and DPAC-D-PyPF_6_ display large Stokes shifts (70–222 nm), solvent-dependent absorptions and emissions, aggregation-induced emission (AIE), red fluorescence in the aggregated state (*λ*_em_ = 600–650 nm), aggregation-promoted photosensitizing ability with the relative singlet-oxygen quantum yields higher than 1.10, and a mitochondria-targeting ability with the Pearson coefficients larger than 0.85. DPAC-D-PyPF_6_ shows absorption maximum at a longer wavelength, slightly redder fluorescence and better photosensitivity as compared to DPAC-PyPF_6_, which consequently leads to the higher photocytotoxicity under the irradiation of white light as a result of the larger π-conjugation.

## 1. Introduction

Photodynamic therapy (PDT), as an emerging method for the treatment of cancer and bacteria, has attracted much attention due to its low invasiveness, toxicity, and drug resistance [1,2,3,4,5,6,7,8,9,10,11], among which, image-guided PDT is attracting increasing attention for its potential in the theranostics. It has been recognized that exploring high-performance luminescent photosensitizers is very important to the development of image-guided therapy. It is reported that as compared to the traditional photosensitizers [12,13,14,15,16,17,18,19], the photosensitizers with aggregation-induced emission (AIE) not only usually have efficient aggregated-state luminescence but also possess the aggregation-boosted generation of ROS. As such, AIE-active photosensitizers have emerged as a new type of fluorescent photosensitizers [10,11,20,21,22,23,24]. Nevertheless, at present, the types of AIE photosensitizers still need to be expanded.

A class of phenazine derivatives with unique multiple emission properties has been developed since 2015. Specifically, such *N*,*N*′-disubstituted-dihydrodibenzo[*a*,*c*]phenazines emit intense blue light in the constrained state, while relatively moderate orange-red fluorescence is emitted in the unconfined state [25,26,27,28,29,30,31,32]. As can be seen from the molecular structure, blue light is the intrinsic emission of dihydrodibenzo[*a*,*c*]phenazines, because the saddle-shaped configuration of the phenazine ring gives rise to the limited π-conjugation. When molecularly dispersed, the dihydrodibenzo[*a*,*c*]phenazines could undergo vigorous intramolecular vibrations and rotations. The active intramolecular motions partially consume the excited-state energy which accounts for the not very efficient emission of dihydrodibenzo[*a*,*c*]phenazines in the unconstrained state. In addition, the intramolecular vibrations of the central phenazine ring render the conformation changing from bent to planar, which leads to the elongation of the π-conjugation and the resultant abnormal orange-red emission. A series of theoretical calculations and experiments have been carried out to prove that the emergence of the orange-red emission is induced by the intramolecular bent-to-planar vibrations. Therefore, such a phenomenon was named vibration-induced emission (VIE) [25]. Thus, it is known that, in the dihydrodibenzo[*a*,*c*]phenazine system, VIE and AIE compatibly coexist. As a result of the structural-flexibility, these dihydrodibenzo[*a*,*c*]phenazines usually show environment-sensitive fluorescence, which can be continuously tuned from orange-red to blue light as the intramolecular motions restrict and vice versa, as a result of the collective effects of VIE and AIE. Such unique photophysical properties make DPAC derivatives a class of excellent ratiometric fluorescent probes with large Stokes shifts [33,34,35,36,37,38,39,40,41,42,43,44,45,46,47,48]. Based on this, these VIE+AIE systems have been widely used in many fields [33,34,35,36,37,38,39,40,41,42,43,44,45,46,47,48]. For example, DPAC-based fluorogens have been used for the detection or recognition of chemical species such as metal cations [33,34], dicarboxylate dianions [35,36], and glucose [37], explored for the sensing of physical parameters such as temperature [38,39], viscosity [40] and moisture [41], and real-time monitoring of the specific processes such as gelation [42], self-assembly [43], and microphase separation [44]. Noteworthily, the DPAC derivatives have also been developed recently for biological applications [45,46,47,48], such as the ratiometric detection and blocking of influenza viruses [46,47], and the in vivo three-photon fluorescence imaging [48]. Undoubtedly, the VIE plus AIE feature endows the dihydrodibenzo[*a*,*c*]phenazines with great charm to be tailored as functional materials.

**Scheme 1:**
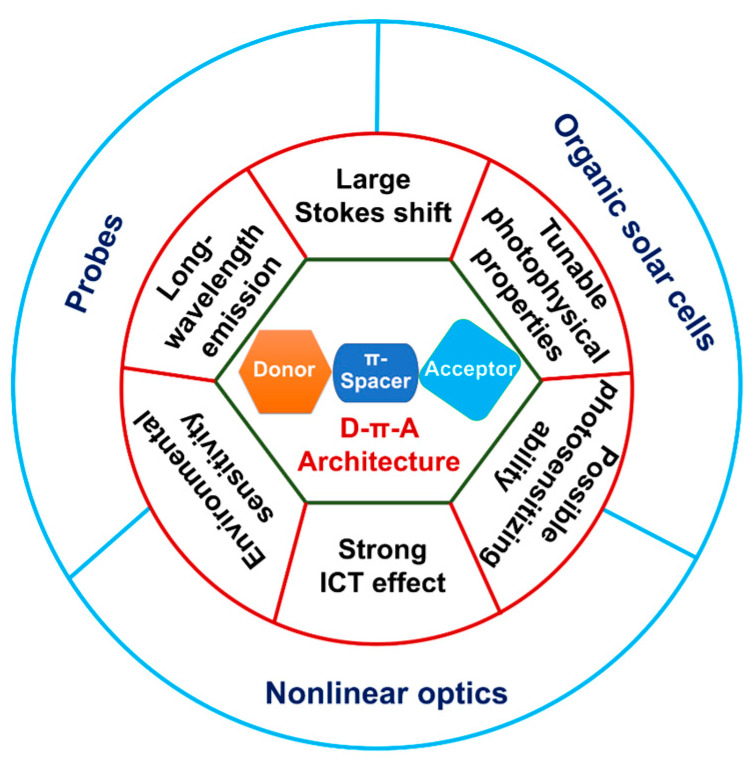
The main characteristics and application areas of D-π-A-structured molecular systems.

**Scheme 2:**
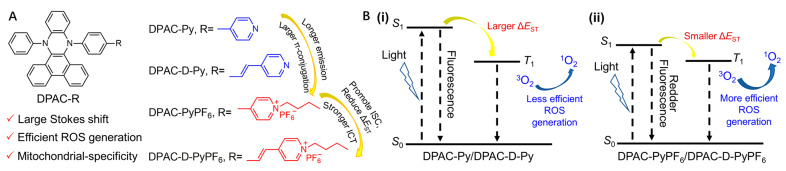
(**A**) Structures of the pyridinium-DPAC adducts DPAC-PyPF_6_ and DPAC-D-PyPF_6_, and their corresponding control compounds DPAC-Py and DPAC-D-Py. (**B**) Schematic illustration of the mechanism of the ROS generation of these DPAC derivatives under light irradiation, where diagrams (**i**,**ii**) represent the excited-state photophysical processes of DPAC-Py/DPAC-D-Py and DPAC-PyPF_6_/DPAC-D-PyPF_6_, respectively.

At the same time, it has been reported that cancer cells are richer in mitochondria compared to normal cells [49]. Therefore, fluorescent photosensitizers with mitochondria-targeting abilities have attracted much attention, because they can ensure the specificity and effectiveness of the photosensitizers in the imaging and therapy of cancer [50,51]. A number of cationic AIEgens have so far been reported to possess mitochondria-specificity. Fluorophores functionalized with pyridinium group(s) have attracted numerous attentions in organelle-specific imaging [52,53,54,55,56], due to the easy introduction of pyridinium units and the positive charge of pyridinium which promotes the probes to target the negatively charged organelle such as mitochondria via electrostatic interactions. Moreover, as reported, the construction of D-π-A architecture would bring about many benefits [57,58,59,60], such as strong ICT effect, large Stokes shift, which is vital to the imaging applications [61,62,63,64], long-wavelength emission, environmental sensitivity, tunable photophysical properties, and even photosensitizing properties, etc. (Scheme 1). Based on the D-π-A structure, a large number of functional materials for applications in fields including probes, organic solar cells, and non-linear optics have been explored. Herein, we intend to introduce positively charged pyridinium unit into the DPAC framework featured with both AIE and VIE characteristics, in the hope of obtaining a D-π-A-structured fluorescent molecular system with efficient red light in the aggregated state, aggregation-promoted photosensitizing ability, and mitochondria-targeting ability. We designed and synthesized pyridinium-modified DPAC derivatives DPAC-PyPF_6_ and DPAC-D-PyPF_6_, comparatively studied their photophysical properties and then elaborated their structure–activity relationship with DPAC-Py and DPAC-D-Py as the control compounds. The theoretical and experimental results show that the ICT originated from the D-π-A structure contributes to the separation of HOMO and LUMO, promoting intersystem crossing (ISC), and thus facilitating the photosensitization. DPAC-PyPF_6_ and DPAC-D-PyPF_6_ both showed large Stokes shifts greater than 200 nm, solvent-dependent absorption and emission, AIE characteristics, efficient aggregated-state red fluorescence, aggregation-boosted photosensitization and mitochondria-specificity which demonstrated their potential for biomedical applications (Scheme 2).

## 2. Results and Discussion

### 2.1. Rational Design and Facile Synthesis of the DPAC Derivatives

In DPAC-PyPF_6_ and DPAC-D-PyPF_6_, the DPAC framework is supposed to function as the AIE plus VIE unit. The introduction of the pyridinium group is for one thing to form D-π-A structure which can enhance the ICT effect, promote the ISC process, reduce the Δ*E*_ST_ and therefore enhance the efficiency of ROS generation, and for another to afford the cationic DPAC derivatives and further achieve the mitochondria-targeting capability. In the meantime, the absorption, emission, photosensitizing ability, and mitochondria-specificity is envisaged to be tuned by the structural modulation. DPAC-PyPF_6_, DPAC-D-PyPF_6_, and their corresponding control compounds DPAC-Py and DPAC-D-Py were facilely synthesized (Scheme S1). The critical intermediate compound DPAC-Br is synthesized referring to the previous literature [65]. DPAC-Py was obtained through the Suzuki coupling reaction between DPAC-Br and the 4-pyridylboronic acid, while the DPAC-D-Py was produced via the Heck reaction of DPAC-Br and the 4-vinylpyridine. Finally, DPAC-PyPF_6_ and DPAC-D-PyPF_6_ were obtained after the salification of the pyridine group in DPAC-Py and DPAC-D-Py, respectively. These compounds were fully characterized by ^1^H NMR, ^13^C NMR, and high-resolution mass spectrometry (HRMS) (Figures S1–S12).

### 2.2. Single-Crystal Structures of the DPAC Derivatives

The single crystals of DPAC-Py and DPAC-PyPF_6_ have been successfully obtained by slowly diffusing hexane into the tetrahydrofuran (THF) solutions of DPAC-Py and DPAC-PyPF_6_, respectively. The detailed structure parameters of these single crystals are summarized in Figures S13 and S14, and Table S1. Similar to the parent structure, namely the DPAC core, the single-crystal structures of DPAC-Py and DPAC-PyPF_6_ suggest that they are both saddle-shaped, and the dihedral angles of the central phenazine ring are 140.48° and 141.05°, respectively (Figures S13 and S14). The torsion angels of DPAC-Py are −39.04°, 19.94° and −24.91°, while those of DPAC-PyPF_6_ are −31.60°, 40.49° and −32.93°, manifesting that they all adopt highly twisted conformations. The greatly distorted conformation can help to avoid the formation of π–π stacking, which can result in efficient emission in the confined states. As suggested by the single-crystal-structure of DPAC-Py, there are four molecules in one unit crystal cell, and multiple intramolecular C–H···π interactions (3.140–3.594 Å), intermolecular C–H···N interactions (3.541 Å and 3.586 Å), and C–H···π interactions (2.692–3.581 Å) between the adjacent molecules. Likewise, in the single-crystal-structure of DPAC-PyPF_6_, there are eight molecules in one unit cell, and the multiple intramolecular C–H···π interactions (3.049–3.570 Å), intermolecular C–H···π interactions (2.698–3.513 Å) and C–H···F interactions (2.395–2.688 Å) can be seen, but no obvious π–π stacking can be observed.

### 2.3. Photophysical Properties of the DPAC Derivatives

#### 2.3.1. Solvatochromic Effect

**Figure 1:**
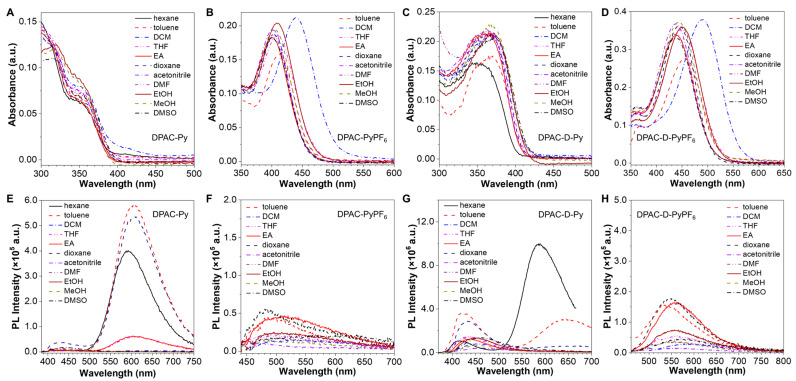
The UV-Vis and fluorescence spectra of (**A**,**E**) DPAC-Py, (**B**,**F**) DPAC-PyPF_6_, (**C**,**G**) DPAC-D-Py and (**D**,**H**) DPAC-D-PyPF_6_ in different solvents, *c* = 10 μM.

In the light of the D-A structures of these DPAC derivatives, especially DPAC-PyPF_6_ and DPAC-D-PyPF_6_, we firstly tested their solvatochromic effect. The absorption maxima of DPAC-Py in different solvents are around 365 nm (Figure 1A), which are consistent with the absorption maximum of DPAC. However, the absorption maxima (*λ*_abs_s) of DPAC-PyPF_6_ in different solvents are longer than 390 nm, suggestive of an obvious bathochromic shift after the salification. As the solvent polarity increases, the *λ*_abs_ red shifts from 398 nm (in ethyl acetate (EA)) to 409 nm (in ethanol (EtOH)). It might be because there is an ICT process between the electron-rich DPAC group and the electron-withdrawing pyridinium group, resulting in the significant bathochromic shift in the *λ*_abs_ with the increasing polarity of solvent (Figure 1B). With the introduction of the vinyl group, the absorption maxima of DPAC-D-Py in different solvents are around 375 nm, which are obviously bathochromic compared to those of DPAC-Py. It is because the vinyl group increases the overall length of π-conjugation (Figure 1C). Similarly, the *λ*_abs_ of DPAC-D-PyPF_6_ is red-shifted as compared to that of DPAC-PyPF_6_, and the *λ*_abs_s of DPAC-D-PyPF_6_ in different solvents are longer than 430 nm. Clearly, after the salification, the *λ*_abs_s of DPAC-D-PyPF_6_ are also bathochromic as compared to those of DPAC-D-Py. With the increase of solvent polarity, the *λ*_abs_ of DPAC-D-PyPF_6_ red-shifts from 438 nm (in EA) to 450 nm (in EtOH), which is might also due to the ICT effect (Figure 1D).

DPAC-Py exhibits red fluorescence in solvents with low polarity (*λ*_em_ ≈ 610 nm) and orange-red fluorescence in the dispersed state (*λ*_em_ = 590–600 nm), which is consistent with the VIE characteristics of the parent DPAC. However, the fluorescence emissions are much weaker in solvents with high polarity as compared to those in the solvents with low polarity, which might be due to the quenching of fluorescence by the ICT effect (Figure 1E). The fluorescence emissions of DPAC-PyPF_6_ in different solvents are very weak. This is possibly because the low-polarity solvent has poor solubility to DPAC-PyPF_6_, which leads to the severe inner-filter effect. As for the situation in high-polarity solvents, the fluorescence quenching is probably due to the strong ICT effect (Figure 1F). DPAC-D-Py exhibits dual emissions, i.e., orange-red and blue fluorescence, in hexane, toluene, and DCM (Figure 1G). It might be because the intramolecular vibrations of the phenazine unit are partially hindered, which leads to the appearance of blue fluorescence. Such a result suggests the more rigid conformation of DPAC-D-Py as compared to DPAC-Py. Meanwhile, DPAC-D-Py merely shows blue but weak fluorescence in other solvents, which might be due to the fluorescence quenching originating from the ICT effect. Like DPAC-PyPF_6_, DPAC-D-PyPF_6_ shows weak fluorescence emission in different solvents (Figure 1H), which might also be ascribed to the intense ICT effect.

#### 2.3.2. AIE/VIE Properties

**Figure 2:**
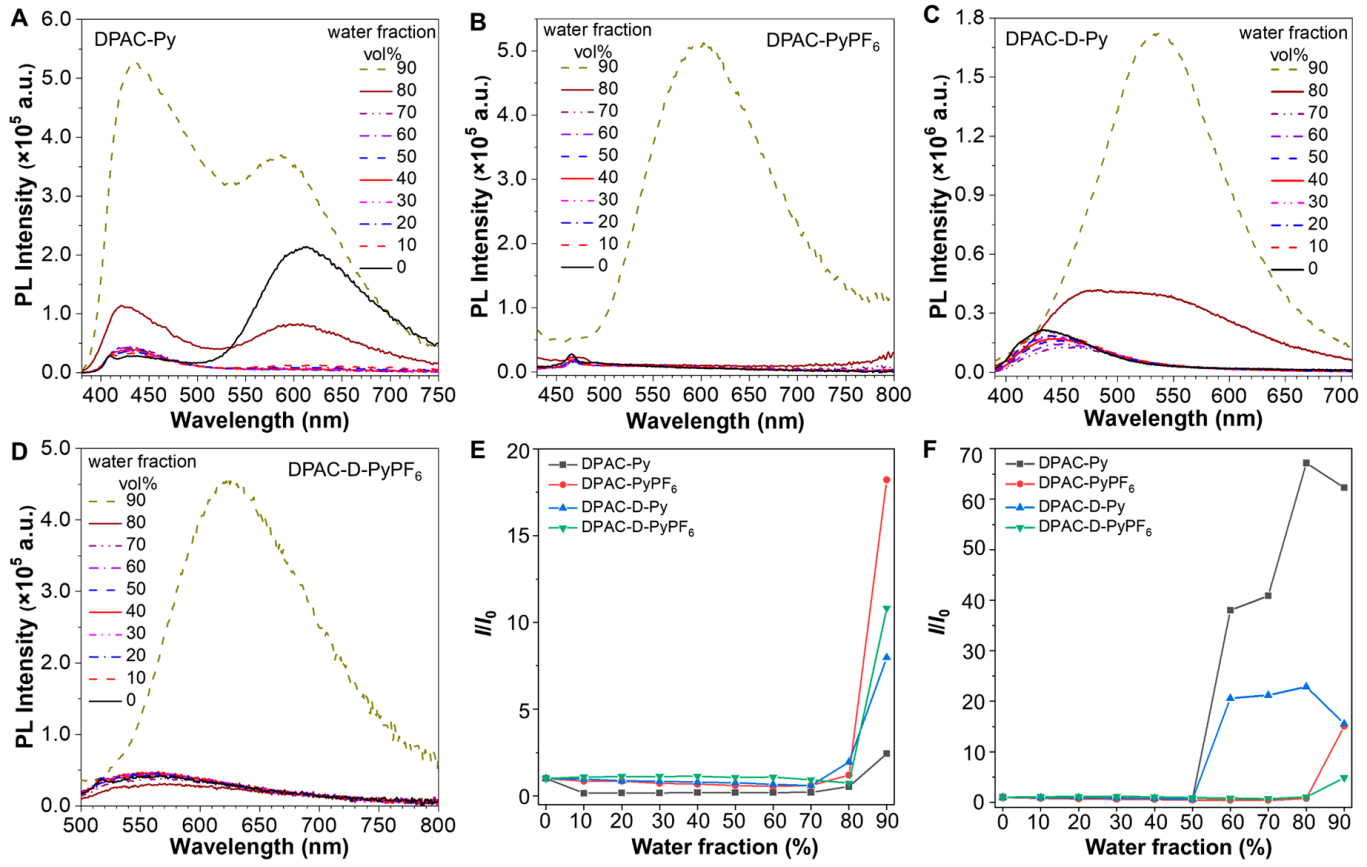
The fluorescence spectra of (**A**) DPAC-Py, (**B**) DPAC-PyPF_6_, (**C**) DPAC-D-Py and (**D**) DPAC-D-PyPF_6_ in the THF/water mixtures with different *f*_w_s at room temperature, *c* = 10 μM, *λ*_ex,A_ = 365 nm, *λ*_ex,B_ = 409 nm, *λ*_ex,C_ = 367 nm, *λ*_ex,D_ = 450 nm. The corresponding plots of the emission enhancement (*I*/*I*_0_) versus *f*_w_s in the (**E**) THF/water, and (**F**) DMSO/water mixtures, respectively.

As discussed above, DPAC is a protype VIE fluorophore and moreover its structure follows the restrictions of the intramolecular motions (RIM) principle, thus we tested the AIE/VIE performance of these four DPAC derivatives. We first investigated their fluorescence behaviors in the classical binary solvent system used for the evaluation of AIE behaviors, i.e., tetrahydrofuran (THF)/water. DPAC-Py exhibits dual emissions in THF before water is added (Figure 2A). After the addition of increasing amounts of water (<80 vol%), the red fluorescence emission disappears first. It might be because the water addition causes the increase in solvent polarity which quenches the red light. When the water content reaches 80 vol%, the orange-red emission reappears again and increases with the blue emission simultaneously. Further enhancement of these dual emissions is achieved when the water content is 90 vol%. The fluorescence enhancement when the water content arrives at 80 vol% might be due to the aggregation which restricts the intramolecular motions and activates the AIE process. DPAC-PyPF_6_ exhibits extremely weak emission in the blue light region THF, but intense orange-red emission in the THF/water mixture when the water content is up to 90% (Figure 2B), suggesting the remarkable AIE effect. Similarly, DPAC-D-Py and DPAC-D-PyPF_6_ merely emit weak blue fluorescence in the THF solution. On the contrary, when the water fraction is 80 vol%, DPAC-D-Py exhibits moderate greenish-blue emission while strong green light when the water fraction (*f*_w_) reaches 90 vol% (Figure 2C). Analogue to DPAC-PyPF_6_, DPAC-D-PyPF_6_ is quite weakly blue-emissive in THF but displays very intense red emission at 90 vol% of water content (Figure 2D). As revealed by the plots of *I*/*I*_0_ versus *f*_w_ depicted in Figure 2E, these four DPAC derivatives are all AIE-active (Figure 2E). The results of the corresponding UV-Vis absorption spectra (Figure S15), dynamic light scattering (DLS, Figure S16) and transmission electron microscope (TEM, Figure S17) further prove the formation of aggregates and the AIE properties (Table 1).

To further demonstrate the AIE properties considering the further bioapplications, their fluorescent behaviors were investigated in the mixtures of DMSO/water with different water fractions. In the DMSO solution, all these DPAC derivatives are very weakly fluorescent. In the DMSO/water system, DPAC-Py exhibits blue fluorescence peaked at 474 nm with the water content increasing from 60 vol% to 80 vol% (Figure S18A). The emission of DPAC-Py at 90 vol% is slightly weaker than that at 80 vol% which might be due to the inner filter effect. As for DPAC-PyPF_6_, it shows an intense orange-red emission with the peak at 603 nm only when the water fraction reached 90 vol% (Figure S18B). When the water content is no more than 80 vol%, the fluorescence of DPAC-PyPF_6_ is also quenched due to the ICT, while when the water content is 90%, DPAC-PyPF_6_ aggregates and the orange-red emission is greatly enhanced. As the water content arrives at 60 vol%, an intense green emission (*λ*_em_ = 517 nm) of DPAC-D-Py emerges and gets slightly enhanced when the water fraction further increases to 70 vol% and then to 80 vol% (Figure S18C). It might be because of the inner filter effect. The fluorescence at 90 vol% becomes lower than 60 vol%. Similar to DPAC-PyPF_6_, DPAC-D-PyPF_6_ almost displays no emission until the water content reaches 90 vol% (Figure S18D), with the fluorescence maximum at 634 nm. As such, the AIE characteristics of these four DPAC derivatives are further reflected by the plots shown in Figure 2F. It can be seen that the high polarities of the DMSO and DMSO/water mixtures exert a remarkable quenching effect on the VIE band of the DPAC moiety. The emission maximum increases from DPAC-Py to DPAC-D-Py to DPAC-PyPF_6_ and then to DPAC-D-PyPF_6_, as a result of the elongation of the π-conjugation and the enhancement of the ICT effect.

To exclude the effect of water, we switched the binary solvent system from aqueous solutions to the THF/hexane mixtures (Figure S19). DPAC-Py and DPAC-PyPF_6_ were used as models for this measurement. The THF solution of DPAC-Py displays weak dual emissions, showing typical VIE effect. With the addition of hexane, DPAC-Py is always dual-emissive, and the orange-red emission at 600 nm increases gradually (Figure S19A). It might be because DPAC-Py is well-dissolved in hexane and the low polarity of hexane inhibits the emission quenching of the VIE band. As for DPAC-PyPF_6_, the case in THF/hexane system is similar to those in THF/water and DMSO/water. It shows almost no emission in the THF/hexane mixture with the hexane fraction lower than 80 vol%, and intensively emits when the hexane fraction reaches 80 vol% (Figure S19B). It is clear that DPAC-PyPF_6_ is insoluble in hexane. Due to the strong ICT effect, the emission of DPAC-PyPF_6_ is very weak in the solution state, while the large amount of hexane added caused aggregation of DPAC-PyPF_6_ and the AIE effect was shown. Moreover, possible binary good/poor solvent systems including nonpolar, polar aprotic, and polar protic solvents were also applied to systematically investigate the photophysical behaviors of DPAC-Py and DPAC-PyPF_6_ (Figures S20–S23). The results further prove the AIE feature and the polarity sensitivity of the VIE band of the DPAC moiety.

#### 2.3.3. Solid-State Emission Behaviors

**Figure 3:**
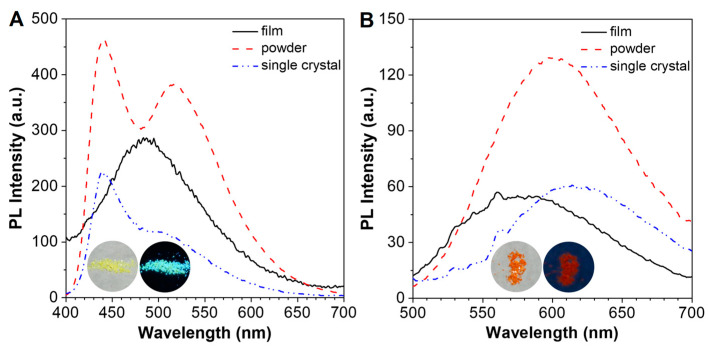

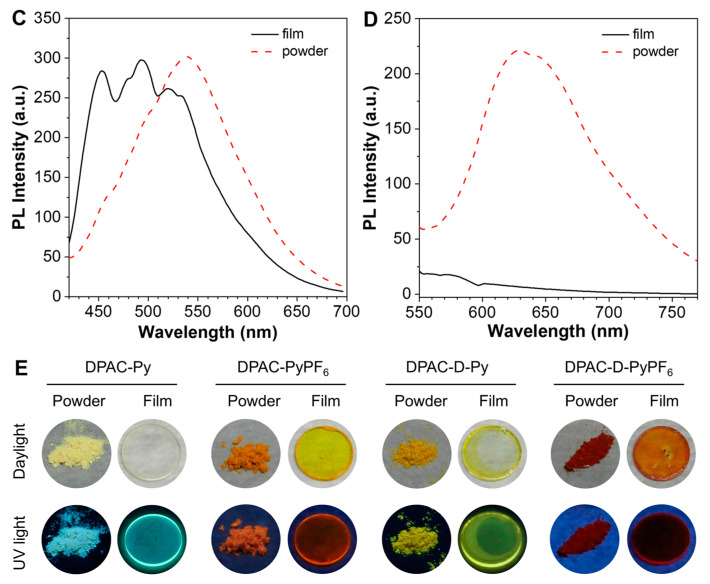
The FL spectra of (**A**) DPAC-Py and (**B**) DPAC-PyPF_6_ as film, powder, and single crystal, (**C**) DPAC-D-Py and (**D**) DPAC-D-PyPF_6_ as film and powder, *λ*_ex,A_ = 365 nm, *λ*_ex,B_ = 401 nm, *λ*_ex,C_ = 370 nm, *λ*_ex,D_ = 450 nm. Insets: The photographs of the single crystals of DPAC-Py and DPAC-PyPF_6_ taken under daylight (left) and UV illumination (right). (**E**) The photographs of DPAC-Py, DPAC-PyPF_6_, DPAC-D-Py, and DPAC-D-PyPF_6_ in the powder and film states taken under daylight and UV light.

Subsequently, we tested the solid-state excitation (Figure S24), absorption (Figure S25), and emission properties (Figure 3A) of these four molecules. The powder of DPAC-Py exhibits a remarkable dual-emission with the blue light (@442 nm) slightly stronger than the green light (@516 nm). The single crystal of DPAC-Py also shows dual-emission, but the blue fluorescence (440 nm) is overwhelmingly more intense than the green light (497 nm). In the thin film state, DAPC-Py fluorescent green light peaked at 490 nm. The differences of the excitation, absorption, and emissions of DPAC-Py at different states are supposed to be associated with the packing mode and compactness. In other words, the more compact the molecular packing, the bluer the light. The DPAC-Py molecules in the single crystal are more compactly packed as compared to those in the powder and film state and hence the corresponding emission is bluer. Moreover, the powders might contain multiple phases, while the film is more homogeneous and hence displays a broad emission band. As for DPAC-PyPF_6_, both the powder and the single crystal mainly exhibit a single red-fluorescence band situated at 602 and 615 nm, respectively (Figure 3B), which is consistent with that of the aggregated state discussed above. The film of DPAC-PyPF_6_ is orange fluorescent with the emission maximum at 585 nm (Figure 3B). The red-shift in the emission maximum of the single crystal and powder of DPAC-PyPF_6_ as compared to its film might be due to the more compact molecular packing which facilitates the intermolecular interaction. The film of DPAC-D-Py is greenish-yellow emissive with multiple shoulder peaks, reflecting the various conformations of DPAC-D-Py in the film state (Figure 3C). The emission maximum of the powders of DPAC-D-Py is at 537 nm and consistent with that of the aggregated state which was mentioned above (Figure 3C). The emission of DPAC-D-PyPF_6_ in the film state is very weak due to its strong ICT effect and relatively loose packing. In contrast, the powder-state emission of DPAC-D-PyPF_6_ is peaked at 631 nm and much stronger than that of the film, which agrees with that of the aggregated state discussed above (Figure 3D). The images of these four compounds at different states taken under daylight and UV light are consistent with the spectral results (Figure 3E and the insets of Figure 3A,B).

### 2.4. Electrochemical Properties and the ICT Effect of the DPAC Derivatives

**Figure 4:**
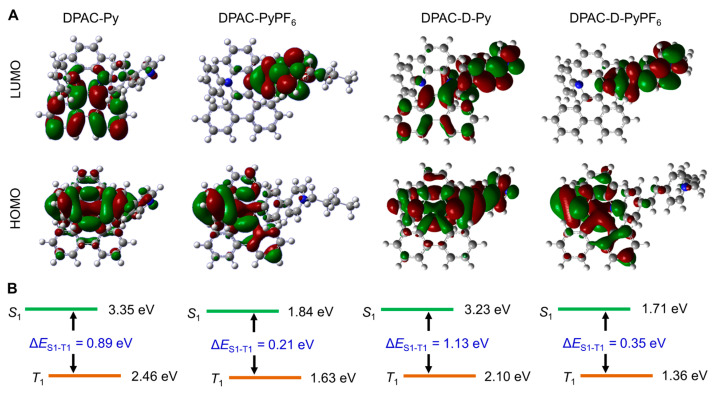
(**A**) HOMO and LUMO and (**B**) the singlet (*S*_1_)-, triplet (*T*_1_)-state energy levels, and the energy gap between *S*_1_ and *T*_1_ (Δ*E*_ST_) of DPAC-Py, DPAC-PyPF_6_, DPAC-D-Py, and DPAC-D-PyPF_6_, respectively, obtained from the DFT calculations.

To fully understand the structure–property relationship of these compounds, the HOMOs and LUMOs of these four compounds were investigated by cyclic voltammetry and the TD-DFT calculations (Figure S26 and Figure 4), with the corresponding results summarized in Table 2. The HOMO energy levels of DPAC-Py, DPAC-PyPF_6_, DPAC-D-Py, and DPAC-D-PyPF_6_ determined by the cyclic voltammetry are around −5.00 eV, while the optical band gaps (*E*_g_) were calculated to be 3.09, 2.46, 2.96 and 2.30 eV, respectively. The *E*_g_s estimated with the equation of *hc*/*λ* are 3.72, 3.18, 3.55, and 2.90 eV, while the ones calculated via TD-DFT method are 3.83, 2.08, 3.52, and 1.90 eV for DPAC-Py, DPAC-PyPF_6_, DPAC-D-Py, and DPAC-D-PyPF_6_, respectively. Obviously, the change of pyridinyl to the pyridinium group narrows the energy gap between HOMO and LUMO. Moreover, the vinyl group in the DPAC-D-Py and DPAC-D-PyPF_6_ results in the elongation of the π-conjugation as compared to DPAC-Py and DPAC-PyPF_6_, and hence gives rise to the decrease in *E*_g_.

The electron clouds of the HOMO of DPAC-Py are mainly distributed on the diphenyl-substituted phenazine unit, and those of the LUMO are mainly situated on the phenanthrene ring, exhibiting a “vertical” intramolecular charge transfer (Figure 4A). The HOMO of DPAC-PyPF_6_ is mainly distributed on the benzene ring, phenazine group, and part of the phenanthrene ring, and the LUMO is mainly distributed on the other benzene ring linked to the phenazine and the pyridinium group, displaying a “horizontal” ICT. Moreover, both the HOMO and LUMO show obvious charge separation, indicating the strong D-A effect in DPAC-PyPF_6_. The electron clouds of the HOMO of DPAC-D-Py are mainly distributed on the central diphenyl-decorated six-membered heterocycle and the 4-vinylpyridine group, while the electron clouds of the LUMO is mainly dispersed on the phenanthrene ring and the 4-vinylpyridine group. It is clear that both the ground state and the excited state of DPAC-D-Py have charge separation, and moreover, when excited, the charges transfer “vertically” from the diphenyl-substituted phenazine group to the phenanthrene ring. It is similar to the case of DPAC-Py. Analogous to the other three DPAC derivatives, DPAC-D-PyPF_6_ also has a remarkable charge transfer, both at the ground state and the excited state. The HOMO of DPAC-D-PyPF_6_ is mainly contributed by the benzene ring, the six-membered heterocyclic ring and part of the phenanthrene ring, while the LUMO is mainly distributed on the other benzene group and 4-vinylpyridinium group. It thus can be seen that quite similar to that of DPAC-PyPF_6_, there is a dramatic “horizontal” ICT in DPAC-D-PyPF_6_. The HOMO and LUMO energy levels were also calculated based on the single crystal structures of DPAC-Py and DPAC-PyPF_6_ and the results are in good agreement with those obtained from the optimized structures (Figure S27). It is worth mentioning that the energy gap between *S*_1_ and *T*_1_ (Δ*E*_ST_) of DPAC-Py, DPAC-PyPF_6_, DPAC-D-Py, and DPAC-D-PyPF_6_ is 0.89, 0.21, 1.13, and 0.35 eV (Figure 4B), respectively, agreeing well with the above discussions. The electrochemical measurements and the DFT calculations have provided good explanations of the photophysical properties.

### 2.5. ROS-Generating and Mitochondria-Targeting Abilities of the DPAC Derivatives

**Figure 5:**
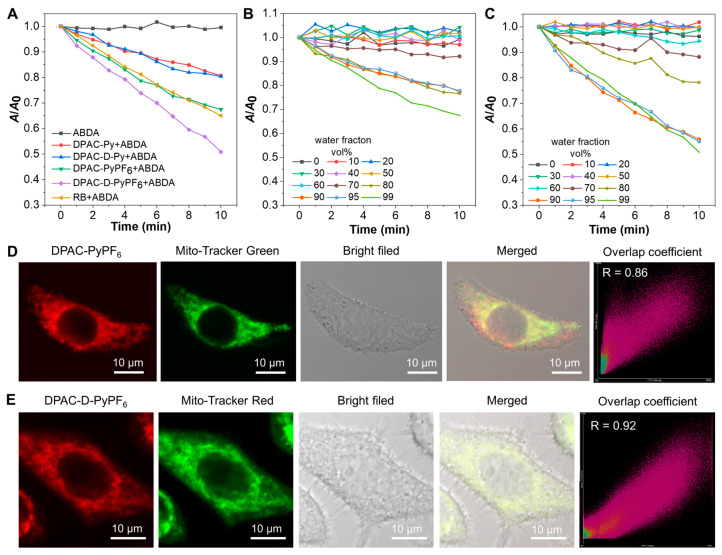
(**A**) The decomposition rates of ABDA only and when ABDA (50 mM) coexists with DPAC-Py, DPAC-D-Py, DPAC-PyPF_6_, DPAC-D-PyPF_6_ (10 mM) or Rose Bengal (10 mM) in the aqueous solution (containing 1% DMSO) under white-light irradiation (10 mW/cm^2^). Rates of ABDA decomposition when the ABDA (50 mM) coexists with (**B**) DPAC-PyPF_6_ (10 mM) or (**C**) DPAC-D-PyPF_6_ (10 mM) in the DMSO/water mixtures with different water fractions under white-light irradiation (10 mW/cm^2^). *A*_0_ and *A* are the absorbance of ABDA at 378 nm before and after white-light irradiation, respectively. The mitochondria-targeting ability of (**D**) DPAC-PyPF_6_ and (**E**) DPAC-D-PyPF_6_ in HeLa cells. *λ*_ex,DPAC-PyPF6_ = 404 nm, *λ*_em,DPAC-PyPF6_ = 570–620 nm, *λ*_ex,MTG_ = 488 nm, *λ*_em,MTG_ = 500–549 nm. *λ*_ex,DPAC-D-PyPF6_ = 404 nm, *λ*_em,DPAC-D-PyPF6_ = 650–700 nm *λ*_ex,MTR_ = 561 nm, *λ*_em,MTR_ = 570–620 nm. [probe] = 5 μM, [MitoTracker] = 250 nM, Scale bar: 10 μm.

It is known that the remarkable ICT effect and the relatively small Δ*E*_ST_ values (Figure 4B) usually imply the ability to generate reactive oxygen species (ROS). In view of this, we used 9,10-anthracene-bis(methylene) dimalonic acid (ABDA) as the ^1^O_2_ indicator to test the ROS-generating ability of these four DPAC derivatives (Figure 5A–C and Figures S28–S30). When the DPAC derivatives are irradiated with white light, the absorbance of ABDA at 378 nm decreases dramatically, suggesting the generation of ^1^O_2_. Compared with the commercial photosensitizer, Rose Bengal (RB), it can be seen that the photosensitizing capability of DPAC-D-PyPF_6_ is better than that of RB, while the photosensitizing abilities of DPAC-Py, DPAC-D-Py, and DPAC-PyPF_6_ are worse than that of RB. The good photosensitizing properties of DPAC-D-PyPF_6_ can be ascribed to the change from pyridine to pyridinium and the introduction of the vinyl group. For one thing, the salification of the pyridine group enhanced the ICT effect and thus promoted the ISC, which is conducive to the ROS production. For another, the vinyl group elongated the π-conjugation length. Due to the strongest ICT and longest π-conjugation, DPAC-D-PyPF_6_ has the longest absorption wavelength, causing it to have the best absorbing ability of white light among all of these four DPAC derivatives. Subsequently, we tested the ROS-producing ability of DPAC-PyPF_6_ and DPAC-D-PyPF_6_ under different aggregated states (Figure 5B,C, Figures S29 and S30). With the increase in water content, their ability to generate ROS was gradually enhanced, indicating that aggregation was conducive to the generation of ROS. The aggregated-state singlet-oxygen quantum yields of DPAC-PyPF_6_ and DPAC-D-PyPF_6_ are measured to be 1.27 and 1.18, respectively (Figures S31 and S32), with RB as the reference. Such data further verified the superior photosensitizing capability of DPAC-PyPF_6_ and DPAC-D-PyPF_6_ over RB.

Considering the positive charge, large Stokes shift, and good photophysical properties of the pyridinium-furnished DPAC derivatives, we assessed their ability to target mitochondria. MitoTracker Green (MTG) and MitoTracker Red (MTR) were selected as the commercial mitochondria indicators for DPAC-PyPF_6_ and DPAC-D-PyPF_6_, respectively (Figure 5D,E andFigure S33). When co-incubated with HeLa cells, DPAC-PyPF_6_ and DPAC-D-PyPF_6_ show excellent ability to target mitochondria, with the Pearson coefficient of 0.86 and 0.92, respectively.

### 2.6. Cytotoxicity of the DPAC Derivatives

**Figure 6:**
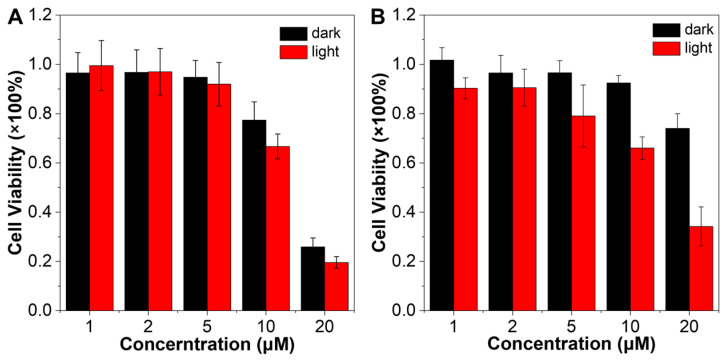
Viability of HeLa cells treated with different concentrations of (**A**) DPAC-PyPF_6_ or (**B**) DPAC-D-PyPF_6_ with and without white-light irradiation (72 mW/cm^2^) for 0.5 h and further incubated for 4 h in dark.

Before the evaluation of the cytotoxicity of DPAC-PyPF_6_ and DPAC-D-PyPF_6_, we first explored their optimal time taken up by the cells (Figures S34 and S35). After co-incubating the probes with HeLa cells for different times (0.5 h, 1 h, 1.5 h, 2 h, 2.5 h, 3 h), the optimal cellular time taken of DPAC-PyPF_6_ and DPAC-D-PyPF_6_ was determined to be 2 h, as indicated by the CLSM results. Subsequently, HeLa cells were incubated with different concentrations of DPAC-PyPF_6_ and DPAC-D-PyPF_6_ to assess the cytotoxicity of these two DPAC derivatives (Figure 6). Under the irradiation of white light, the cell survival rate decreases with the increase in the concentration of DPAC-PyPF_6_ and DPAC-D-PyPF_6_. It is worth noting that the dark cytotoxicity of these two compounds, especially DPAC-PyPF_6_ is unignorable. The photocytotoxicity of DPAC-D-PyPF_6_ is higher as compared to that of DPAC-PyPF_6_. It is because, as shown in Figure 5A, DPAC-D-PyPF_6_ has higher ROS generation capability than DPAC-PyPF_6_. Moreover, the longer absorption wavelength and better absorption of white light of DPAC-D-PyPF_6_ also contribute to the photocytotoxicity. Pitifully, although the ROS-generation efficiency of DPAC-PyPF_6_ and DPAC-D-PyPF_6_ is quite high, their photocytotoxicity is not satisfactory enough. It might be because of the relatively poor cell-penetration ability of white light, or due to the relatively short absorption wavelengths and the inefficient absorption of the white light of DPAC-PyPF_6_ and DPAC-D-PyPF_6_. Nevertheless, combing their mitochondrial-targeting ability, dark cytotoxicity and photocytotoxicity, both DPAC-PyPF_6_ and DPAC-D-PyPF_6_ have a potential to be used as image-guided dual-therapeutic agents which show the chemotherapy and photodynamic therapy effect.

## 3. Materials and Methods

### 3.1. Chemicals and Instruments

All reagents and chemicals used in this work were purchased from commercial suppliers and used without further purification. DPAC-Br was prepared following the previously reported procedures [65]. Mitotracker^®^ Green FM and Mitotracker^®^ Red FM were purchased from Beyotime Biological Company (Shanghai, China). The ^1^H NMR and ^13^C NMR spectra were measured by Bruker AM-400 spectrometer (Bruker Technology GmbH, Karlsruhe, Baden-Württemberg, Germany), with 0.03% tetramethylsilane (TMS) as the internal reference and CDCl_3_ or DMSO-*d*_6_ as the solvent. The high-resolution mass spectrometry (HRMS) data were obtained by Waters LCT premier XE spectrometer (Waters Co., Ltd., Milford, MA, USA) and Xevo G2 TOF MS (Waters Co., Ltd., Milford, MA, USA). The UV-Vis absorption spectra were measured by an Agilent Cary 60 UV-Vis spectrometer (Agilent Technologies, Santa Clara, CA, USA), using 1 cm quartz cells. The photoluminescence spectra except the AIE properties evaluation were recorded in 1 cm quartz cells on a fluorescence spectrophotometer (Agilent Cary Eclipse (Agilent Technologies, Santa Clara, CA, USA) or Horiba Fluoromax 4 (HORIBA Scientific, Piscataway, NJ, USA)). The solid-state absorbance spectra were recorded on Shimadzu UV-2600 (Shimadzu, Kyoto, Japan). The AIE measurements were conducted using 1 cm quartz cells on Shimadzu RF-6000 (Shimadzu, Kyoto, Japan). The absolute fluorescence quantum yields were determined on Horiba Fluoromax-4 (HORIBA Scientific, Piscataway, NJ, USA). Dynamic light scatting (DLS) experiments were performed on Zetasizer Nano ZSE (Marvin Panalytical Ltd., Worcestershire, UK). Transmission electron microscopy (TEM) images were obtained by a JEM-1400 biological transmission electron microscope (JEOL Ltd., Tokyo, Japan). The cell imaging experiments were conducted on a Nikon A1R confocal laser scanning microscope (CLSM) (Nikon Corporation of Japan, Tokyo, Japan). The X-ray crystallographic measurements of DPAC-Py and DPAC-PyPF_6_ were carried out on Bruker D8 Venture (Bruker Technology GmbH, Karlsruhe, Baden-Württemberg, Germany), with the MoK*α* (*λ* = 0.71073 Å) and CuK*α* (*λ* = 1.54178 Å) as radiation source for DPAC-Py and DPAC-PyPF_6_, respectively. The results were further analyzed with SHELXL-2013 (Simens (Bruker), Göttingen, Germany).

### 3.2. Synthesis

The synthetic procedures of DPAC-Py are as follows. The mixture of DPAC-Br (500 mg, 0.976 mmol), pyridine-4-boronic acid (360 mg, 2.929 mmol), K_2_CO_3_ (1.935 g, 14.000 mmol), tetrakis(triphenylphosphine)palladium (56 mg, 0.0485 mmol), tetrahydrofuran (THF, 28 mL), and water (7 mL) were put into a two-necked round-bottomed flask. The mixture was kept refluxing with vigorous stirring for 12 h under an atmosphere of nitrogen. After cooling down, the mixed solution was extracted three times with dichloromethane (DCM) and water to obtain a black oily substance. The crude product was purified through column chromatography on silica gels (petroleum ether (PE)/ethyl acetate (EA) = 4/1, *v*/*v*) to afford a light green solid (yield: 250 mg, 50.1%). ^1^H NMR (400 MHz, CDCl_3_) *δ* 8.76 (dd, *J* = 8.3, 3.4 Hz, 2H), 8.51 (s, 2H), 8.11 (d, *J* = 8.2 Hz, 2H), 7.84–7.73 (m, 2H), 7.68 (td, *J* = 8.1, 1.2 Hz, 2H), 7.62–7.51 (m, 2H), 7.43–7.29 (m, 6H), 7.08–6.97 (m, 6H), 6.82–6.74 (m, 1H). ^13^C NMR (100 MHz, CDCl_3_) *δ* 149.91, 148.62, 147.92, 147.46, 145.24, 144.15, 138.73, 137.58, 130.20, 130.03, 129.63, 129.45, 129.32, 129.00, 127.77, 127.57, 127.47, 127.30, 127.22, 126.94, 126.85, 126.02, 125.56, 124.88, 124.53, 123.33, 123.23, 121.47, 120.83, 117.11, 116.20. HRMS (ESI-TOF, *m*/*z*): [M]^+^ calcd. for C_37_H_25_N_3_: 512.2127; found: 512.2121.

The synthetic procedures of DPAC-PyPF_6_ are as follows. The mixture of DPAC-Py (100 mg, 0.196 mmol), 1-iodobutane (1.78 mL (2.848 g), 15.650 mmol) and THF (3 mL) was put into a two-necked round-bottomed flask. The mixture was kept refluxing with vigorous stirring for 4 h under an atmosphere of nitrogen. Yellow solids were precipitated in the solution. After cooling down, the mixture was filtered and washed with THF for several times to collect the yellow solids. The mixture of yellow solids, saturated KPF_6_ solution (3 mL), and dimethyl sulfoxide (DMSO, 10 mL) were put into another flask. The mixture was kept at 100 °C with vigorous stirring for 2 h under the atmosphere of nitrogen. After cooling down, a large amount of water was added to the flask, and orange-red solids were precipitated out. The mixture was filtered and washed several times with water to collect the orange-red solids (yield: 80 mg, 57.3%). ^1^H NMR (400 MHz, DMSO-*d*_6_) *δ* 9.00 (dd, *J* = 8.2, 5.5 Hz, 2H), 8.90 (d, *J* = 7.0 Hz, 2H), 8.28 (d, *J* = 7.0 Hz, 2H), 8.09–7.93 (m, 4H), 7.88 (d, *J* = 9.1 Hz, 2H), 7.83–7.60 (m, 4H), 7.58–7.46 (m, 2H), 7.14–7.01 (m, 6H), 6.84 (dt, *J* = 8.2, 2.0 Hz, 1H), 4.47 (t, *J* = 7.3 Hz, 2H), 1.93–1.73 (m, 2H), 1.37–1.16 (m, 2H), 0.90 (t, *J* = 7.4 Hz, 3H). ^13^C NMR (100 MHz, DMSO-*d*_6_) *δ* 153.47, 149.93, 146.56, 144.28, 144.08, 142.21, 137.83, 135.77, 129.78, 129.36, 129.29, 129.10, 128.01, 127.77, 127.66, 127.54, 127.42, 127.21, 127.13, 126.68, 125.79, 124.58, 124.05, 123.93, 123.83, 123.44, 122.56, 121.87, 117.34, 115.40, 59.17, 32.44, 18.69, 13.28. HRMS (ESI-TOF, *m*/*z*): [M–PF_6_]^+^ calcd. for C_41_H_34_N_3_^+^: 568.2753; found: 568.2768.

The synthetic procedures of DPAC-D-Py are as follows [65]. The mixture of DPAC-Br (500 mg, 0.976 mmol), 4-vinylpyridine (0.21 mL (0.210 g), 1.950 mmol), K_2_CO_3_ (163 mg, 1.175 mmol), palladium acetate (22 mg, 0.098 mmol), and DMF (40 mL) were put into a two-necked round-bottomed flask. The mixture was kept refluxing with vigorous stirring for 24 h under an atmosphere of nitrogen. After cooling down and the removal of *N*,*N*′-dimethylformamide (DMF) under reduced pressure, the mixed solution was extracted three times with DCM and water to obtain a black oily substance. The crude product was purified through column chromatography on silica gels (PE/EA = 2/1, *v*/*v*) to afford a yellow solid (yield: 303 mg, 57.8%). ^1^H NMR (400 MHz, DMSO-*d*_6_) *δ* 8.96 (d, *J* = 8.2 Hz, 2H), 8.47 (d, *J* = 5.7 Hz, 2H), 8.02–7.89 (m, 4H), 7.78–7.68 (m, 2H), 7.63 (dd, *J* = 16.0, 8.1 Hz, 2H), 7.49–7.40 (m, 4H), 7.35 (dd, *J* = 12.6, 7.9 Hz, 3H), 7.13–6.90 (m, 7H), 6.82 (t, *J* = 7.0 Hz, 1H). ^13^C NMR (100 MHz, CDCl_3_) δ 149.30, 148.09, 147.35, 145.73, 145.06, 144.03, 138.53, 137.46, 133.32, 130.02, 129.86, 129.32, 129.17, 128.86, 128.33, 127.87, 127.57, 127.44, 127.10, 126.73, 125.83, 125.40, 124.71, 124.41, 123.12, 122.82, 121.31, 120.64, 116.92, 115.84. HRMS (NSI–TOF, *m*/*z*): [M]^+^ calcd. for C_39_H_28_N_3_: 538.2278; found: 538.2263.

The synthetic procedures of DPAC-D-PyPF_6_ are as follows. The mixture of DPAC-D-Py (100 mg, 0.1861 mmol), 1-iodobutane (1.69 mL (2.704 g), 14.694 mmol), and THF (3 mL) were put into a two-necked round-bottomed flask. The mixture was kept refluxing with vigorous stirring for 4 h under an atmosphere of nitrogen. Yellow solids were precipitated out in the solution. After cooling down, the solution was filtered and washed with THF for several times to collect the orange-red solids. The mixture of orange-red solids, saturated KPF_6_ solution (3 mL), and DMSO (10 mL) were put into another flask. The mixture was kept at 100 °C with vigorous stirring for 2 h under an atmosphere of nitrogen. After cooling down, a large amount of water was added to the flask, and red solids were precipitated out. The mixture was filtered and washed several times with water to collect red solids (yield: 65 mg, 47.2%). ^1^H NMR (400 MHz, DMSO-*d*_6_) *δ* 8.99 (d, *J* = 6.9 Hz, 2H), 8.83 (d, *J* = 6.3 Hz, 2H), 8.09 (d, *J* = 6.4 Hz, 2H), 7.98 (d, *J* = 8.0 Hz, 4H), 7.89–7.57 (m, 5H), 7.47 (d, *J* = 8.3 Hz, 4H), 7.19 (d, *J* = 16.1 Hz, 1H), 7.06 (dt, *J* = 17.5, 8.5 Hz, 6H), 6.84 (t, *J* = 6.7 Hz, 1H), 4.43 (t, *J* = 7.2 Hz, 2H), 1.93–1.76 (m, 2H), 1.28 (dd, *J* = 14.6, 7.2 Hz, 2H), 0.91 (t, *J* = 7.3 Hz, 3H). ^13^C NMR (100 MHz, DMSO-*d*_6_) *δ* 153.08, 148.62, 146.78, 144.30, 143.85, 142.80, 140.61, 137.69, 136.22, 129.69, 129.43, 129.38, 129.03, 128.11, 127.95, 127.53, 127.46, 127.38, 127.25, 127.12, 126.38, 125.78, 123.92, 123.82, 123.76, 123.55, 123.13, 121.74, 120.25, 117.27, 115.62, 59.22, 32.42, 18.75, 13.29. HRMS (ESI-TOF, *m*/*z*): [M–PF_6_]^+^ calcd. for C_43_H_36_N_3_^+^: 594.2909; found: 594.2907.

### 3.3. Density Functional Theory (DFT) Calculations

The optimized geometries and the energy levels of the DPAC derivatives were obtained with the density functional theory (DFT) method and a B3LYP/6-31+G* basis set using the Gaussian 09 package. The theoretical calculations based on the single-crystal structures of DPAC-Py and DPAC-PyPF_6_ were implemented with a RB3LYP/6-31G(d) basis set using the Gaussian 09 package [66].

### 3.4. Cyclic Voltammetry Experiments

The cyclic voltammetry experiments were conducted on a CH Instruments Model 1200B (CH Instruments, Inc., Austin, TX, USA) electrochemical workstation using a conventional three-electrode configuration. The glassy carbon, Pt wire, and calomel in the saturated KCl solution were applied as a working electrode, counter electrode, and reference electrode, respectively. The redox potentials were measured in the THF solution containing 1.0 mM of the DPAC derivative and 0.1 M tetra-*n*-butylammonium hexafluorophosphate (TBAPF_6_) as the supporting electrolyte. The cyclic voltammetry grams were recorded at a scanning rate of 0.1 V/s after deoxygenizing the solution with nitrogen.

### 3.5. Reactive Oxygen Species (ROS) Detection in Solution

The 9,10-Anthracenediyl-bis(methylene)-dimalonic acid (ABDA, 50 μM) and a DPAC derivative (10 μM) were added into 10 mL of DMSO/water (*v*/*v* = 1/99) mixture. Upon irradiation with white light for different time periods, the absorption spectra of ABDA were measured. The relative absorption intensity (*A*/*A*_0_) at 378 nm versus irradiation time was then recorded. Besides, the ROS-generating capability of Rose Bengal (RB) which is used as the control was tested under the same experimental conditions. Here, ABDA works as a singlet oxygen indicator.

Similarly, ABDA (50 μM) and a DPAC derivative (10 μM) were added into 10 mL of DMSO/water mixtures with different water fractions to evaluate the photosensitizing ability of the DPAC derivatives in the DMSO/water mixtures with different water fractions. Upon irradiation with white light for different time periods, the absorption spectra of ABDA were measured. The relative absorption intensity (*A*/*A*_0_) at 378 nm versus irradiation time was then recorded.

### 3.6. Singlet-Oxygen Quantum Yield Measurement

In order to eliminate the inner filter effect, the maximum absorbance needs to be around 0.2 a.u. The measurements were carried out under white-light irradiation (10 mW/cm^2^) in DMSO/water (*v*/*v*) = 1/99. Singlet oxygen quantum yields of these AIEgens were calculated by the equation: *Φ*_AIEgen_ = *Φ*_RB_·*K*_AIEgen_·*A*_RB_/(*K*_RB_·*A*_AIEgen_). where *K*_AIEgen_ and *K*_RB_ represent the decomposition rate constants of ABDA in the presence of AIEgen and RB, respectively. *A*_AIEgen_ and *A*_RB_ represent the light absorbance of AIEgen and RB, respectively, which are determined by the integrated areas of the corresponding absorption bands in the wavelength range of 400–800 nm. *Φ*_RB_ is the ^1^O_2_ quantum yield of RB, which is 0.75 in water.

### 3.7. Cell Culture and Fluorescence Imaging

HeLa cells were cultured at 37 °C in DMEM supplemented with 10% fetal bovine serum and 1% penicillin-streptomycin in humidified environment with 5% CO_2_. Cells were seeded on confocal cuvettes and allowed to adhere for 12 h. For cellular uptake experiments, cells were incubated with DPAC-PyPF_6_ or DPAC-D-PyPF_6_ (5 μM or 10 μM) for different times (0.5 h, 1 h, 1.5 h, 2 h, 2.5 h, 3 h). After being washed with PBS (2 mL × 2 times), cell imaging was carried out by a laser confocal scanning microscope. DPAC-PyPF_6_: *λ*_ex_ = 404 nm, *λ*_em_ = 570–620 nm; DPAC-D-PyPF_6_: *λ*_ex_ = 404 nm, *λ*_em_ = 570–620 nm.

For the colocalization experiments, cells were incubated with 5 μM DPAC-PyPF_6_ for 30 min. After being washed with PBS (1 mL × 2 times), cells were incubated with MitoTracker^®^ Green FM (250 nM) for 30 min. Finally, cell imaging was carried out by laser confocal scanning microscope after being washed with PBS (1 mL × 2 times). MitoTracker^®^ Green FM: *λ*_ex_ = 488 nm, *λ*_em_ = 500–550 nm; DPAC-PyPF_6_: *λ*_ex_ = 404 nm, *λ*_em_ = 570–620 nm. Cells were incubated with DPAC-D-PyPF_6_ (5 μM) for 30 min. After being washed with PBS (1 mL × 2 times), cells were incubated with MitoTracker^®^ Red FM (250 nM) for 30 min. Finally, cell imaging was carried out on a confocal laser scanning microscope after being washed with PBS (1 mL × 2 times). MitoTracker^®^ Red FM: *λ*_ex_ = 561 nm, *λ*_em_ = 570–620 nm; DPAC-D-PyPF_6_: *λ*_ex_ = 404 nm, *λ*_em_ = 650–700 nm.

### 3.8. Cytotoxicity Assay

Approximately 10^4^ of HeLa cells were added into each hole of the 96-well plate, and after 12 h incubation, DPAC-PyPF_6_ or DPAC-D-PyPF_6_ of different concentrations (0, 1, 2, 5, 10, 20 μM) were added and incubated for another 2 h. After being exposed to white light (72 mW/cm^2^) for 0.5 h, these cells were further incubated in the dark for 4 h. At the same time, for the dark-cytotoxicity evaluation, the cells in 96-well plates were treated with different concentrations (0, 1, 2, 5, 10, 20 μM) of DPAC-PyPF_6_ or DPAC-D-PyPF_6_ and incubated for 4 h under dark conditions. CCK-8 was then added, and the cells were incubated for another 2 h. The absorbance value was measured at 450 nm by a microplate reader.

## 4. Conclusions

In summary, novel fluorescent photosensitizers have been developed based on the typical VIE system. These photosensitizers are constructed with the *N*,*N*′-disubstituted-dihydrodibenzo[*a*,*c*]phenazines (DPAC) as the skeleton. The pyridine, 4-vinylpyridinyl, pyridinium, and 4-vinylpyridinium groups were introduced onto the DPAC core to tune the ICT effect and π-conjugation length. As a result, the D-π-A-structured DPAC derivatives, namely DPAC-Py, DPAC-D-Py, DPAC-PyPF_6_ and DPAC-D-PyPF_6_, were obtained, and the luminescence, photosensitizing, and mitochondrial-targeting ability were finely modulated. As revealed by the systematic experimental investigation and theoretical calculations, when compared to DPAC-Py and DPAC-D-Py, DPAC-PyPF_6_ has stronger ICT effect while DPAC-D-PyPF_6_ has both stronger ICT effect and longer π-conjugation. Consequently, DPAC-PyPF_6_ and DPAC-D-PyPF_6_ show AIE properties and red fluorescence with large Stokes shifts in the aggregated states, which is different from the VIE phenomenon of the DPAC parent. The cationic DPAC derivatives, i.e., DPAC-PyPF_6_ and DPAC-D-PyPF_6_, are mitochondria-specific with Pearson coefficients of up to 0.86 and 0.92. DPAC-PyPF_6_ displayed a satisfactory ROS generation efficiency and DPAC-D-PyPF_6_ enjoyed ROS-generating ability higher than the other three DPAC derivatives and even higher than that of the commercial photosensitizer Rose Bengal. Moreover, DPAC-PyPF_6_ and DPAC-D-PyPF_6_ show distinct photocytotoxicity and dark cytotoxicity which endows them with the potential to be applied in the mitochondria-targeting and image-guided dual therapy. Thus, this work not only provides a new type of AIE-active photosensitizers but also expands the application scope of DPAC systems to the field of theranostics.

## Figures and Tables

**Table 1:** The photophysical properties of the DPAC derivatives.

		DPAC-Py	DPAC-PyPF_6_	DPAC-D-Py	DPAC-D-PyPF_6_
*λ*_ex_/nm	film	383	449	386/411	455
	powder	387/411	487	387/425	463
	crystal	386/414	500	/	/
*λ*_abs_/nm	solution *^a^*	351	410	367	450
	aggregate *^b^*	365	401	384	437
	film	363	418	378	449
	powder	375	416	383	469
	crystal	393	410/530	/	/
*λ*_em_/nm	solution *^a^*	425/610	N.D.	434	N.D.
	aggregate *^b^*	435/587	603	535	627
	film	490	585	435/493/520	530/608
	powder	442/516	602	537	631
	crystal	440/497	615	/	/
Δ*λ*·*^c^*/nm	aggregate *^b^*	70/222	202	151	190
*ε*·*^d^*/L·mol^−1^·cm^−1^	aggregate *^b^*	3.209 × 10^4^	2.343 × 10^4^	4.462 × 10^4^	2.665 × 10^4^
Φ_F_/%	solution *^a^*	0.11	0	0	0
	aggregate *^b^*	0.34	0.20	0.32	0.12
	powder	8.06	0.24	3.12	0.21

*^a^* Determined with THF solution (10^−5^ M). *^b^* THF/water mixture with a water fraction of 90% (10^−5^ M). N.D.: Not detected. Δ*λ* = Stokes shift. *^c^* Δ*λ* = *λ*_em_−*λ*_abs_. *^d^ ε* = *A*/(*c*·*L*), where *A* = absorbance, *c* = 10^−5^ M, *L* = 1 cm.

**Table 2:** The HOMO and LUMO levels, and the energy gaps of the DPAC derivatives.

Comp.	*E*_g_ *^a^*/eV	*E*_g_ *^b^*/eV	HOMO *^c^*/eV	LUMO *^d^*/eV	HOMO *^e^*/eV	LUMO *^e^*/eV	*E*_g_ *^e^*/eV
DPAC-Py	3.72	3.09	−4.98	−1.89	−5.56	−1.73	3.83
DPAC-PyPF_6_	3.18	2.46	−4.97	−2.51	−7.56	−5.48	2.08
DPAC-D-Py	3.55	2.96	−5.00	−2.04	−5.45	−1.93	3.52
DPAC-D-PyPF_6_	2.90	2.30	−4.97	−2.67	−7.41	−5.51	1.90

*^a^* Estimated from the absorption spectra with the equation of *E* = *hc*/*λ* = (1303.98 nm/*λ*) eV. *^b^* Estimated from onset of the absorption spectra (*E*_g_ = 1241/*λ*_onset_). *^c^* Obtained from the onset of the oxidation voltages and the equation: *E*_HOMO_ = −*e* (*E*_onset,ox_ + 4.4 V). *^d^* Obtained from the equation: *E*_LUMO_ = *E*_HOMO_ + *E*_g_. *^e^* Values obtained from DFT calculation. Comp. = Compound.

## Data Availability

The data presented in this work are available in the paper. The CCDC 2285517 and 2285518 contain supplementary crystallographic data for this paper. These data can be obtained free of charge via http://www.ccdc.cam.ac.uk/conts/retrieving.html (accessed on 29 July 2023) (or from the Cambridge Crystallographic Data Centre, 12, Union Road, Cambridge CB2 1EZ, UK; Fax: +44-1223-336033).

## Supplementary Materials

The following supporting information can be downloaded at: https://www.mdpi.com/article/10.3390/molecules28176392/s1. Scheme S1: The synthetic route to the DPAC derivatives; Figures S1–S12: the spectra of the characterization of the DPAC derivatives; Figures S13 and S14: The crystal structure analysis of DPAC-Py and DPAC-PyPF_6_; Table S1: Crystal data and details of collection and refinement for DPAC-Py and DPAC-PyPF_6_; Figures S15–S17: The UV-Vis spectra, DLS, and TEM analysis of the DPAC derivatives in the THF/water mixtures with different water fractions; Figure S18: The fluorescence spectra of the DPAC derivatives in the DMSO/water mixtures with different *f*_w_s; Figures S19–S23: The fluorescence spectra of the DPAC derivatives in the THF/hexane mixtures with different hexane fractions (*f*_h_s) or in the DCM/hexane mixtures with different *f*_h_s or in the MeOH/water mixtures with different *f*_w_s or in the MeOH/toluene mixtures with different *f*_T_s or in the DMSO/toluene mixtures with different *f*_T_s; Figure S24: The solid-state excitation spectra of the DPAC derivatives; Figure S25: The solid-state absorption spectra of the DPAC derivatives; Figure S26: Cyclic voltammograms of the DPAC derivatives; Figure S27: The HOMOs and LUMOs of DPAC-Py and DPAC-PyPF_6_ calculated by DFT simulation based on the single-crystal structures; Figure S28: Absorption spectra of ABDA in the presence or absence of the photosensitizers at different irradiation time; Figures S29 and S30: Absorption spectra of DPAC-PyPF_6_ or DPAC-D-PyPF_6_ with ABDA in the mixtures of DMSO/water with different water fractions under white-light irradiation for different time; Figures S31 and S32: The measurement of the singlet-oxygen quantum yield; Figure S33: Evaluation of the mitochondria-targeting ability of DPAC-PyPF_6_ or DPAC-D-PyPF_6_; Figures S34 and S35: The CLSM of DPAC-PyPF_6_ or DPAC-D-PyPF_6_ incubated with HeLa cells at 5 μM or 10 μM for different time.

## Sample Availability

Samples of the compounds DPAC-Py, DPAC-D-Py, DPAC-PyPF_6_, DPAC-D-PyPF_6_ are available from the authors.
