# Supplementary material for: Modulating the Luminescence, Photosensitizing Properties, and Mitochondria-Targeting Ability of D-π-A-Structured Dihydrodibenzo[a,c]phenazines
Source: Molecules. 2023 Sep 1;28(17):6392. doi: 10.3390/molecules28176392 (PMC10490149; doi:10.3390/molecules28176392)
Supplement: Supplementary file 1 [file molecules-28-06392-s001.zip › molecules-2563056-supplementary.pdf]

Supporting Information

Modulating the Luminescence, Photosensitizing Properties, and Mitochondria-Targeting Ability of D-π-A-Structured Dihydrodibenzo[*a*,*c*]phenazines

Zhaozhi Zhang [1], Qijing Wang [1], Xinyi Zhang [1], Dong Mei [2,*] and Ju Mei [1,*]

1. Key Laboratory for Advanced Materials, Feringa Nobel Prize Scientist Joint Research Center, Frontiers Science Center for Materiobiology and Dynamic Chemistry, Joint International Research Laboratory for Precision Chemistry and Molecular Engineering, Institute of Fine Chemicals, School of Chemistry and Molecular Engineering, East China University of Science & Technology, 130 Meilong Road, Shanghai, 200237, China.
2. Clinical Research Center, Beijing Children's Hospital, Capital Medical University, National Center for Children's Health, Beijing, 100045, China.

*Correspondence: meidong11290926@126.com (D.M.); daisymeiju@ecust.edu.cn (J.M.)

Synthesis of the DPAC Derivatives

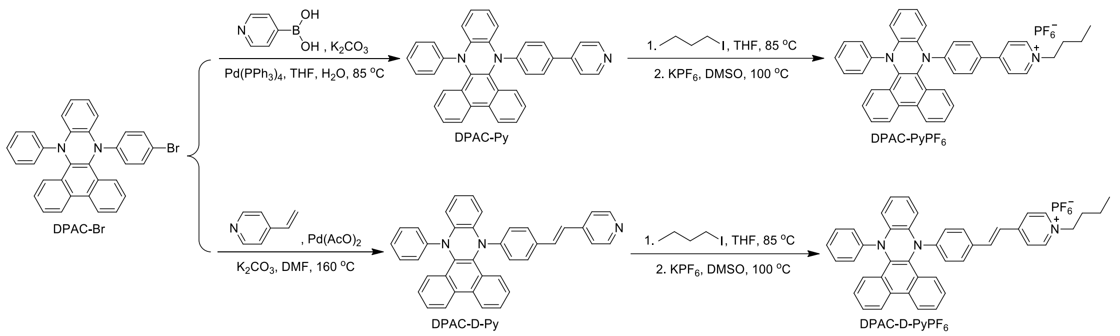

Scheme S1. The synthetic routes of DPAC derivatives.

Structure Characterization of the DPAC Derivatives

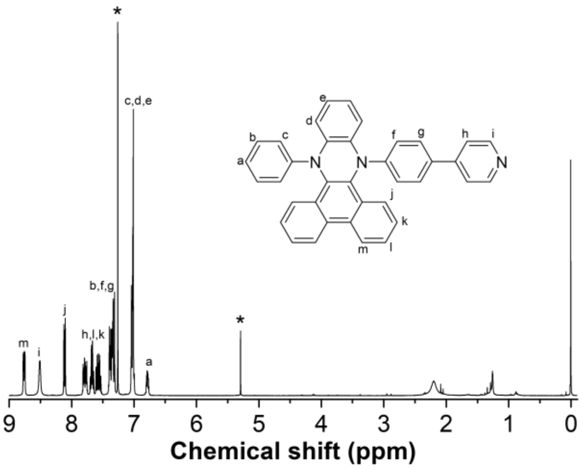

Figure S1. ^{1}H NMR spectrum of DPAC-Py in $CDCl_3$. The solvent peaks are marked with asterisk.

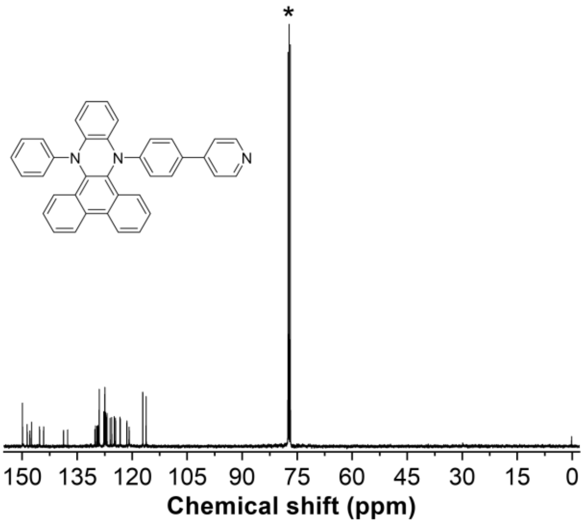

Figure S2. ^{13}C NMR spectrum of DPAC-Py in $CDCl_3$. The solvent peak is marked with asterisk.

Single Mass Analysis
Tolerance = 5.0 PPM / DBE: min = -1.5, max = 50.0
Element prediction: Off
Number of isotope peaks used for i-FIT = 2

Monoisotopic Mass, Even Electron Ions
1 formula(e) evaluated with 1 results within limits (up to 50 best isotopic matches for each mass)
Elements Used:
C: 0-37 H: 0-26 N: 0-3
J-MEI
MJ-ZZZ-005 25 (0.267) Cm (25:26)

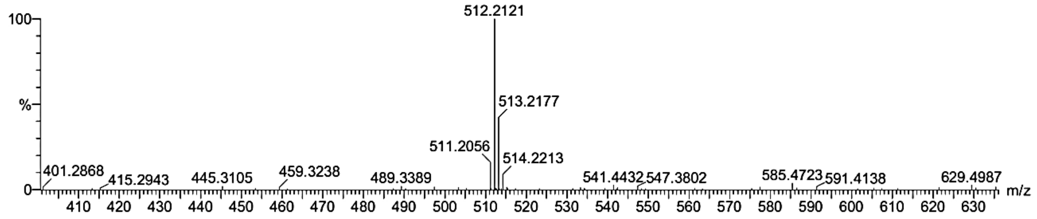

Minimum:				-1.5			
Maximum:		5.0	5.0	50.0			
Mass	Calc. Mass	mDa	PPM	DBE	i-FIT	i-FIT (Norm)	Formula
512.2121	512.2127	-0.6	-1.2	26.5	40.4	0.0	C37 H26 N3

Figure S3. HRMS of DPAC-Py.

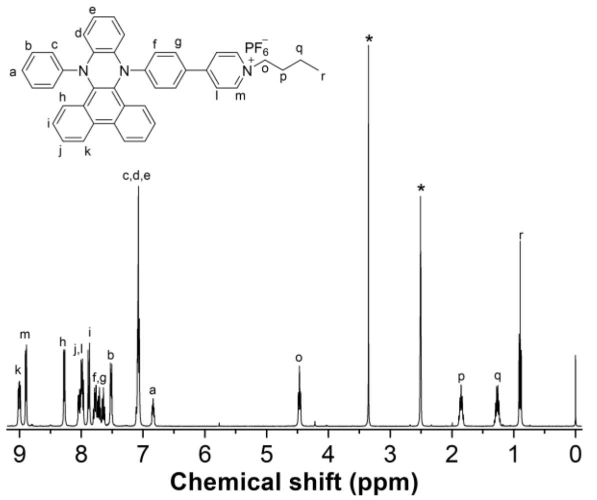

Figure S4. ^{1}H NMR spectrum of DPAC-PyPF$_6$ in DMSO-d_6. The solvent peaks are marked with asterisk.

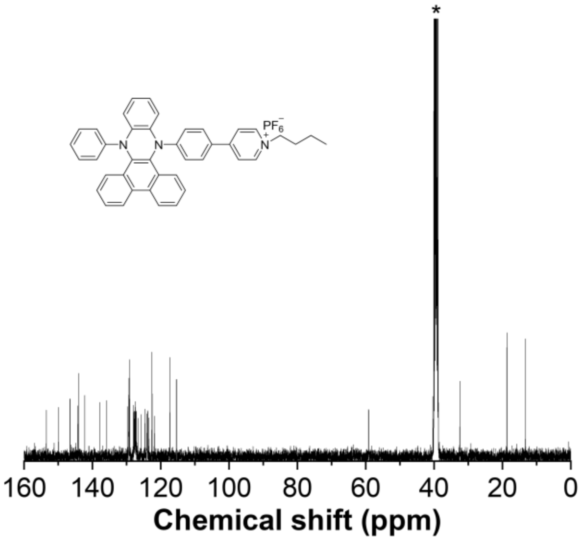

Figure S5. ^{13}C NMR spectrum of DPAC-PyPF$_6$ in DMSO-d_6. The solvent peaks are marked with asterisk.

Single Mass Analysis
Tolerance = 20.0 PPM / DBE: min = -1.5, max = 50.0
Element prediction: Off
Number of isotope peaks used for i-FIT = 3

Monoisotopic Mass, Even Electron Ions
1 formula(e) evaluated with 1 results within limits (up to 50 best isotopic matches for each mass)
Elements Used:
C: 0-41 H: 0-34 N: 0-3
J-MEI
MJ-ZZZ-006 43 (0.480) Cm (43:49)

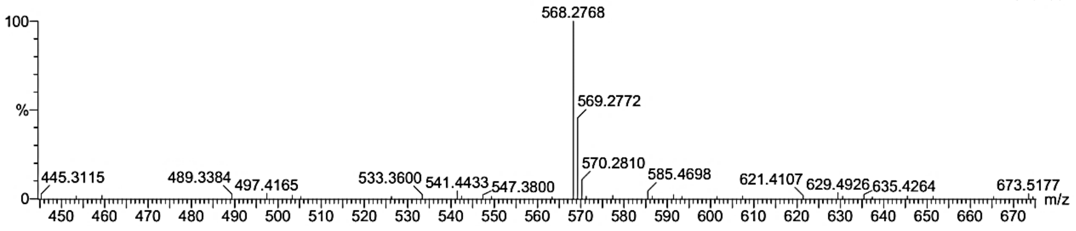

Minimum:				-1.5			
Maximum:		5.0	20.0	50.0			
Mass	Calc. Mass	mDa	PPM	DBE	i-FIT	i-FIT (Norm)	Formula
568.2768	568.2753	1.5	2.6	26.5	14.6	0.0	C41 H34 N3

Figure S6. HRMS of DPAC-PyPF$_6$.

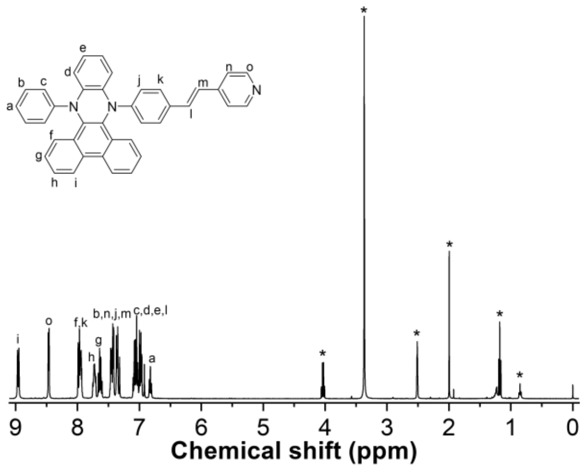

Figure S7. ^{1}H NMR spectrum of DPAC-D-Py in DMSO-d_6. The solvent peaks are marked with asterisk.

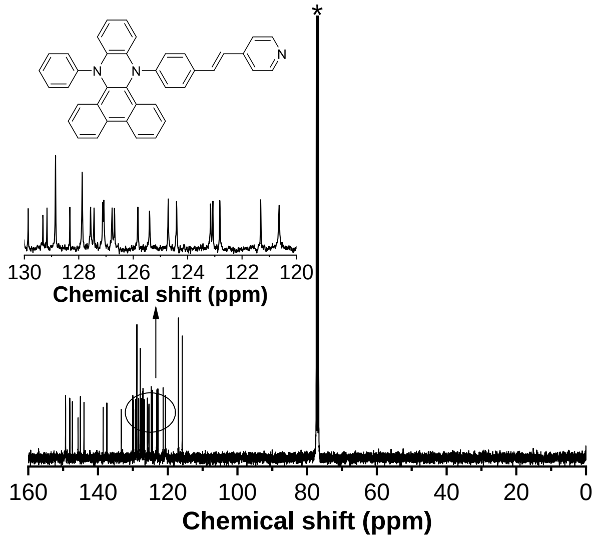

Figure S8. ^{13}C NMR spectrum of DPAC-D-Py in $CDCl_3$. The solvent peaks are marked with asterisk.

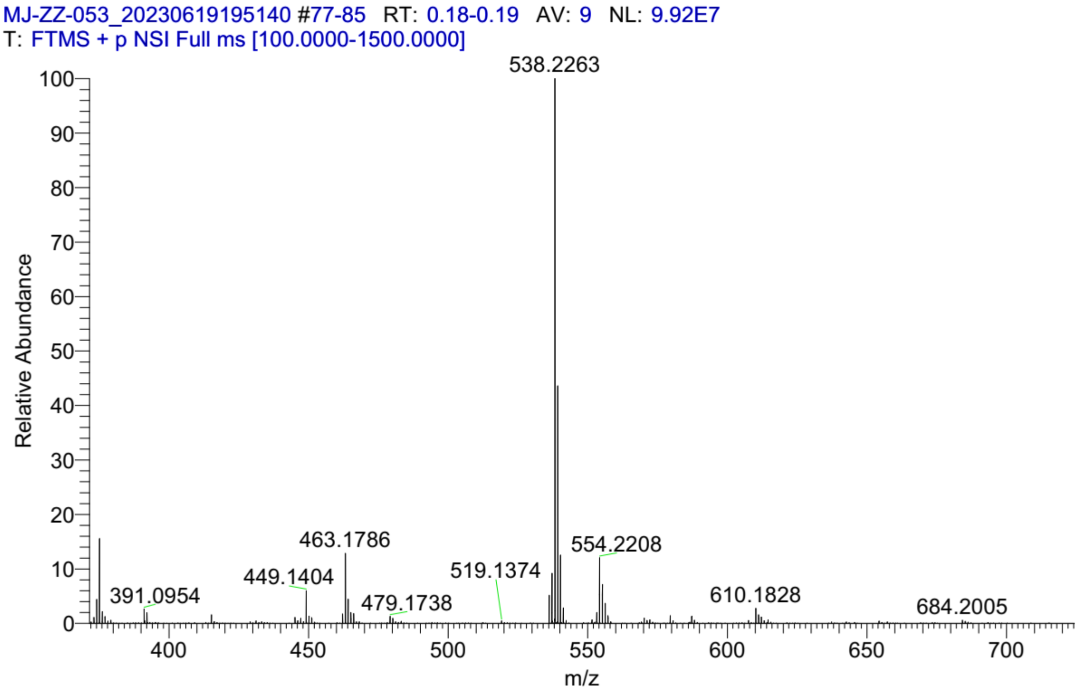

MJ-ZZ-053_20230619195140#76-84 RT: 0.17-0.19 AV: 9
T: FTMS + p NSI Full ms [100.0000-1500.0000]
m/z= 536.93-538.75

m/z	Intensity	Relative	Theo. Mass	Delta (ppm)	Composition
538.2263	106530992.0	100.00	538.2278	-1.43	$C_{39}H_{28}N_3$

Figure S9. HRMS of DPAC-D-Py.

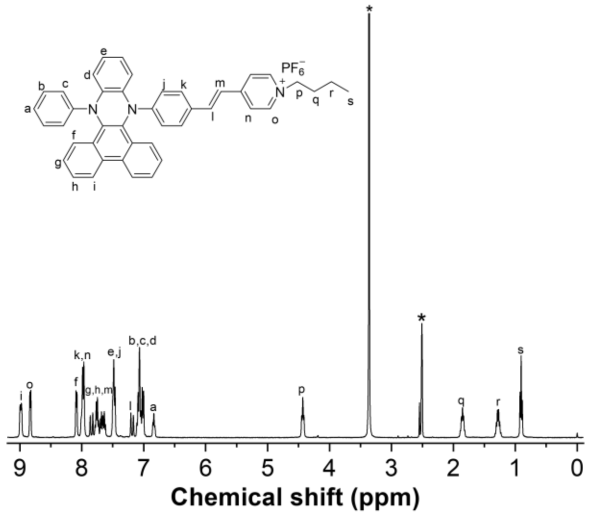

Figure S10. ^{1}H NMR spectrum of DPAC-D-PyPF$_6$ in DMSO-d_6. The solvent peaks are marked with asterisk.

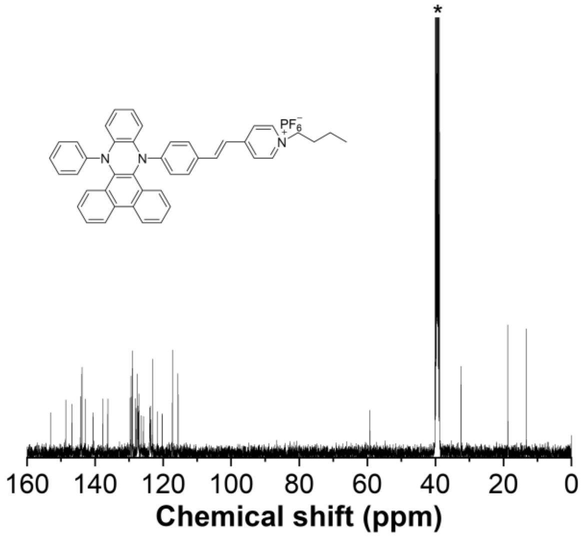

Figure S11. ^{13}C NMR spectrum of DPAC-D-PyPF$_6$ in DMSO-d_6. The solvent peaks are marked with asterisk.

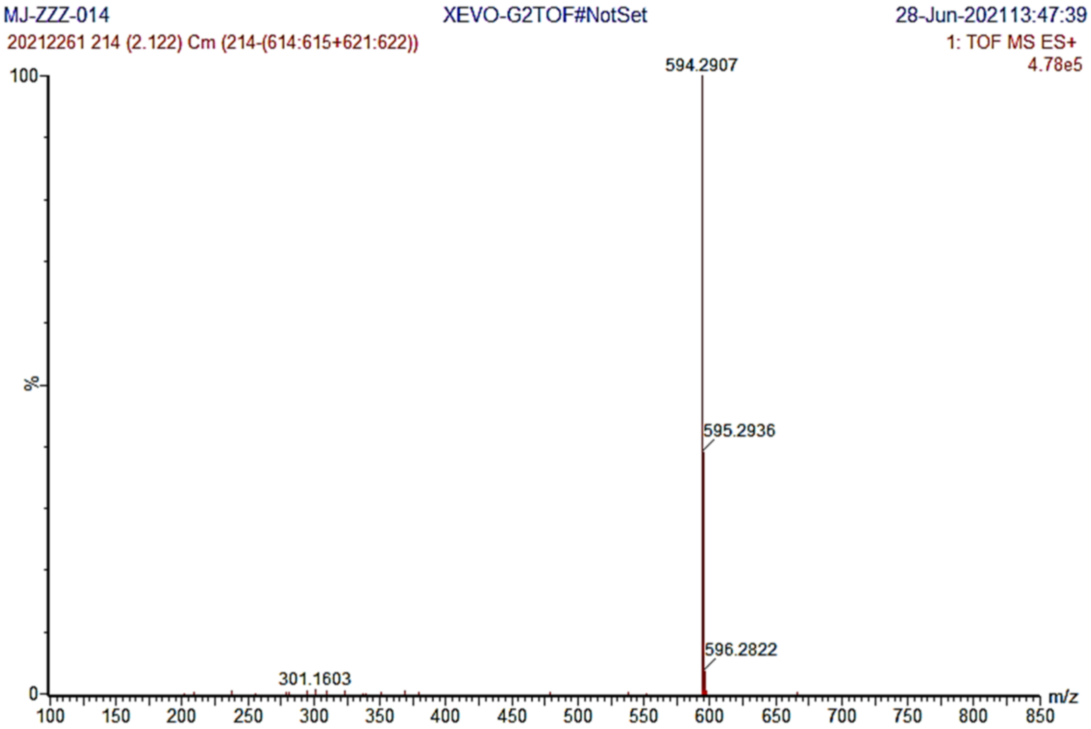

Minimum				–1.5			
Maximum		20.0	5.0	50.0			
Mass	Calc. Mass	mDa	PPM	DBE	i-FIT	i-FIT (Norm)	Formula
594.2907	594.2909	–0.2	–0.3	27.5	142.3	2.303	C43 H36 N3

Figure S12. HRMS of DPAC-D-$PyPF_6$.

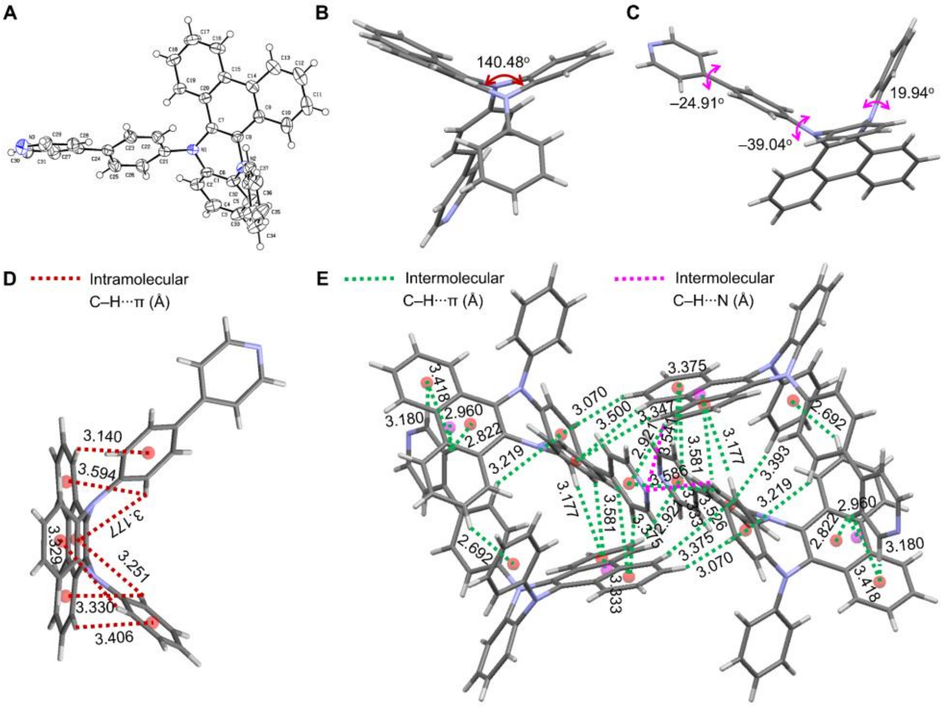

Figure S13. The (A) ORTEP drawings, (B) dihedral angles, (C) torsion angles, (D) intramolecular C–H···π interactions and (E) intermolecular C–H···π and C–H···N interactions of DPAC-Py.

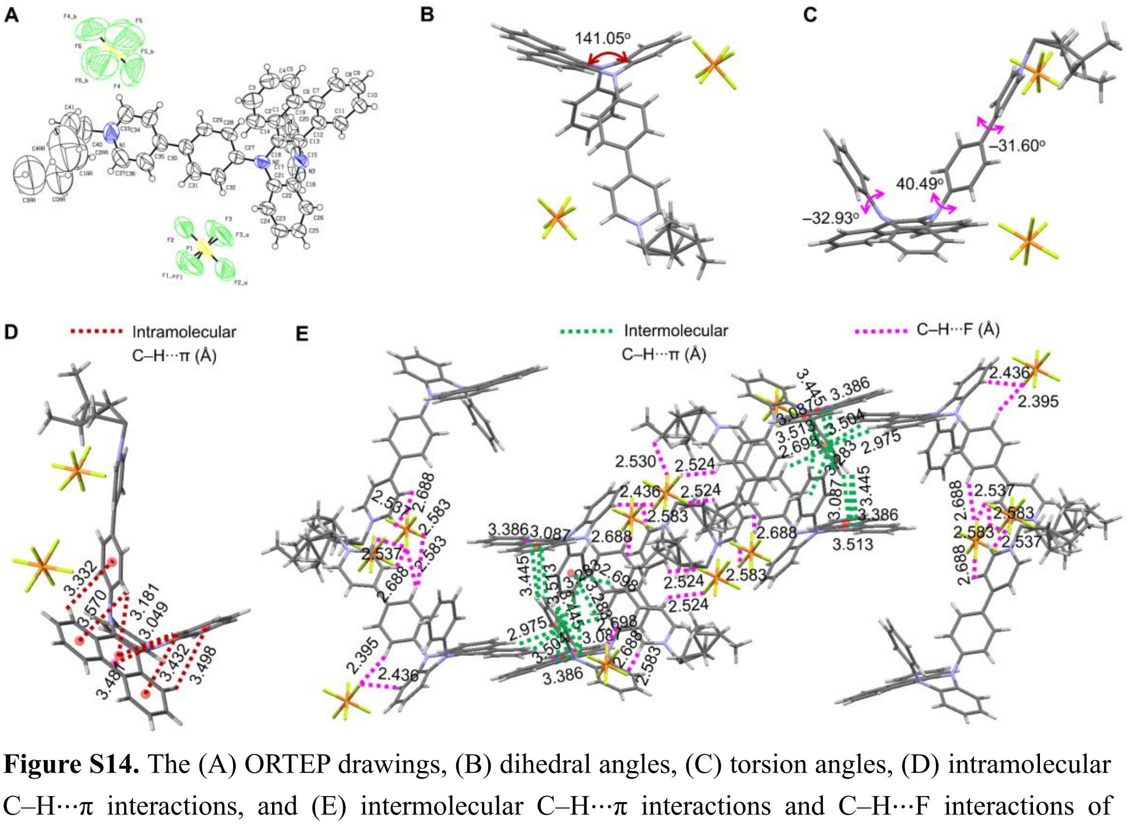

Figure S14. The (A) ORTEP drawings, (B) dihedral angles, (C) torsion angles, (D) intramolecular C–H···π interactions, and (E) intermolecular C–H···π interactions and C–H···F interactions of DPAC-PyPF_6.

Table S1. Crystal data and details of collection and refinement for the DPAC derivatives.

	DPAC-Py	DPAC-$PyPF_6$
CCDC number	2285517	2285518
Empirical formula	$C_{37}H_{25}N_3$	$C_{41}H_{32}N_3PF_6$
Formula weight	511.60	711.66
Temperature (K)	193(2)	173(2)
Crystal system	Monoclinic	Monoclinic
Space group	P 21/n	C 2/c
Cell parameters	a = 15.7882(6) Å	a = 15.9162(4) Å
	b = 10.2161(4) Å	b = 44.9256(9) Å
	c = 16.7011(6) Å	c = 11.6426(3) Å
	α = 90°	α = 90°
	β = 104.8310(10)°	β = 105.056(1)°
	γ = 90°	γ = 90°
Volume [$Å^3$]	2604.04(17)	8039.2(3)
Z	4	8
D_{calcd} [mg/m^3]	1.305	1.176
F (000)	1072	2944
θ range [°]	2.359–25.995	3.936–68.421
Index ranges	–19<=h<=19	–19<=h<=19
	–12<=k<=12	–53<=k<=54
	–20<=l<=20	–14<=l<=14
R1 (all data)	0.0749	0.1459
wR2 (all data)	0.1417	0.4441
R_1 [I > 2σ(I)]	0.0500	0.1303
wR_1 [I > 2σ(I)]	0.1220	0.4160
GOF	1.048	1.951
R (int)	0.0863	0.0511
No. of reflections collects	26600	70903
No. of reflections	5115	7351

Photophysical Properties of the DPAC Derivatives

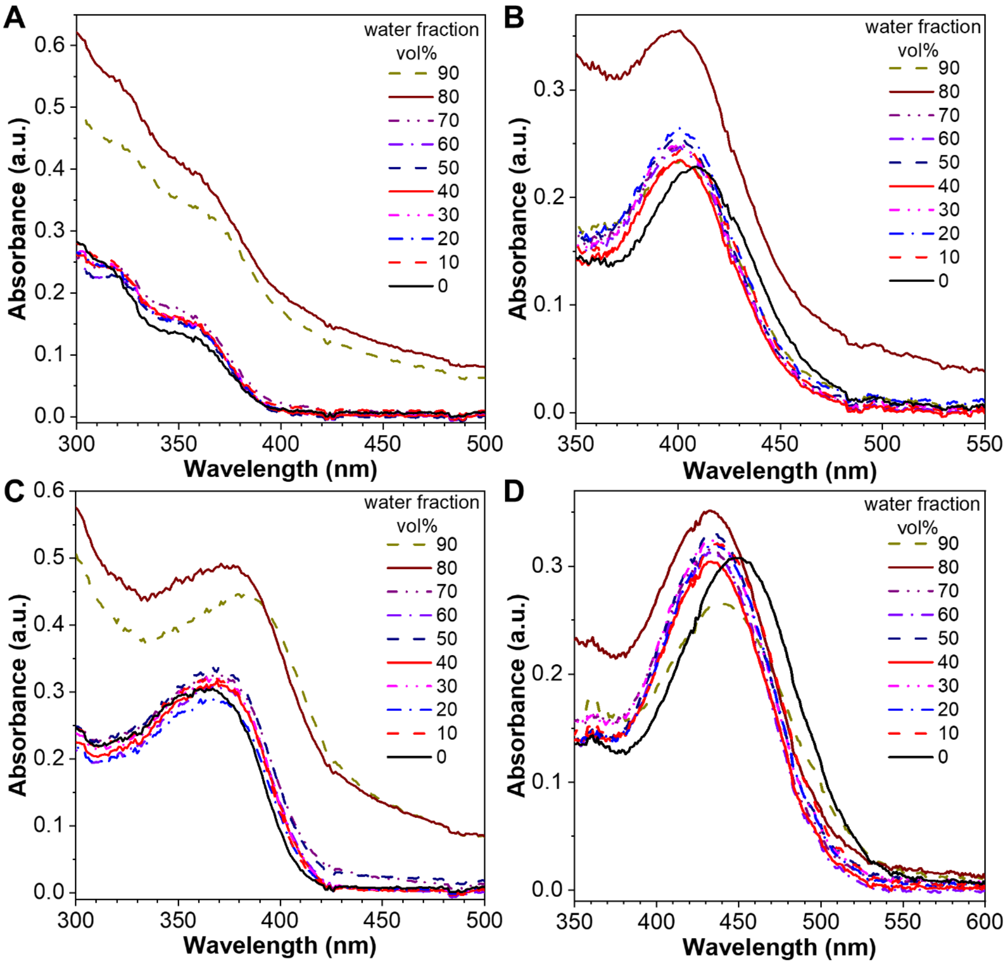

Figure S15. The UV-Vis spectra of (A) DPAC-Py, (B) DPAC-PyPF_6, (C) DPAC-D-Py and (D) DPAC-D-PyPF_6 in the THF/water mixtures with different water fractions (f_ws) at room temperature, c = 10 μM.

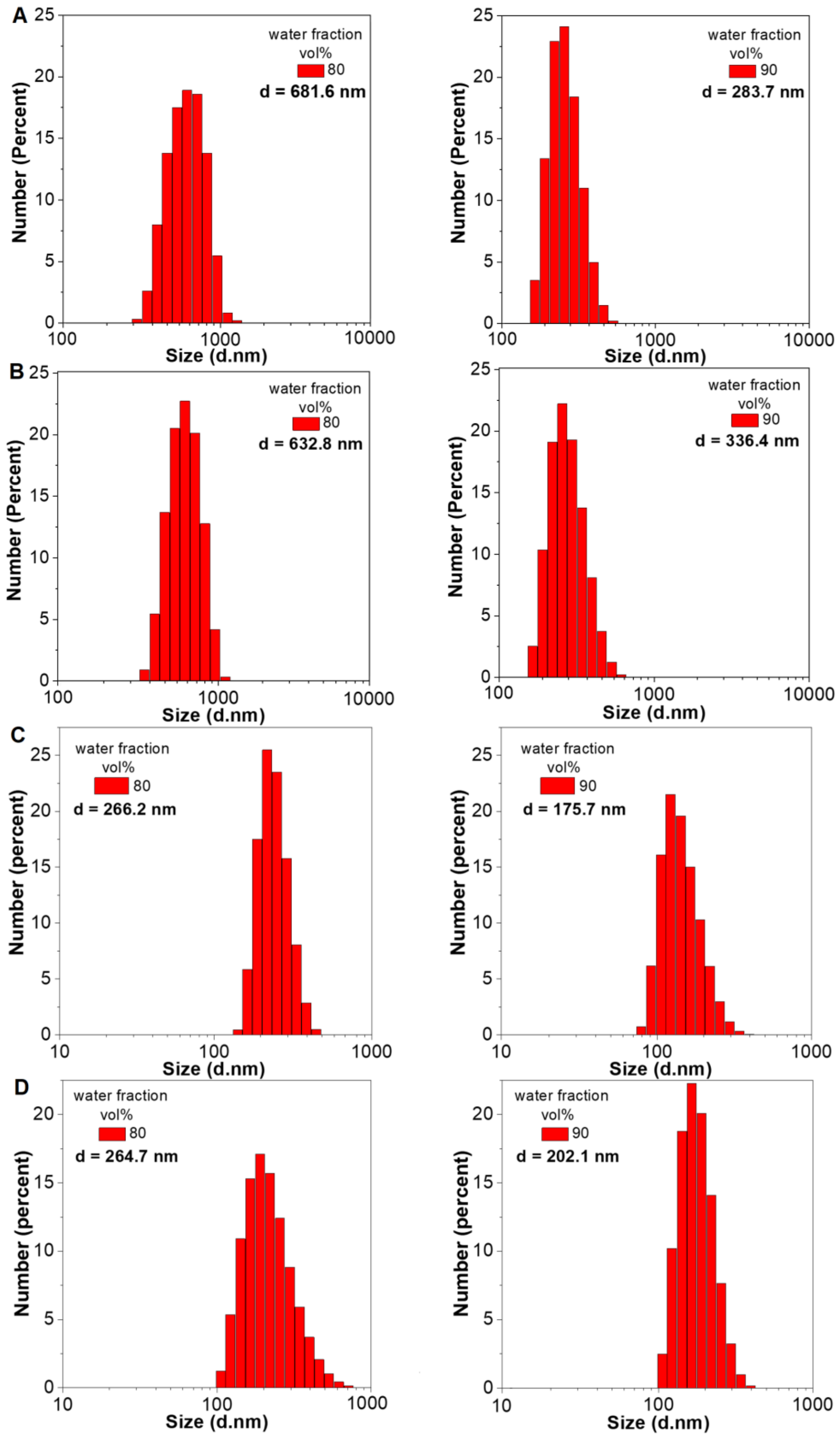

Figure S16. The DLS results of (A) DPAC-Py, (B) DPAC-$PyPF_6$, (C) DPAC-D-Py and (D) DPAC-D-$PyPF_6$ in the THF/water mixtures with different f_ws at room temperature, c = 10 μM. Note: The valid DLS data of these four DPAC derivatives in the THF/water mixtures with f_w lower than 70% can hardly be obtained.

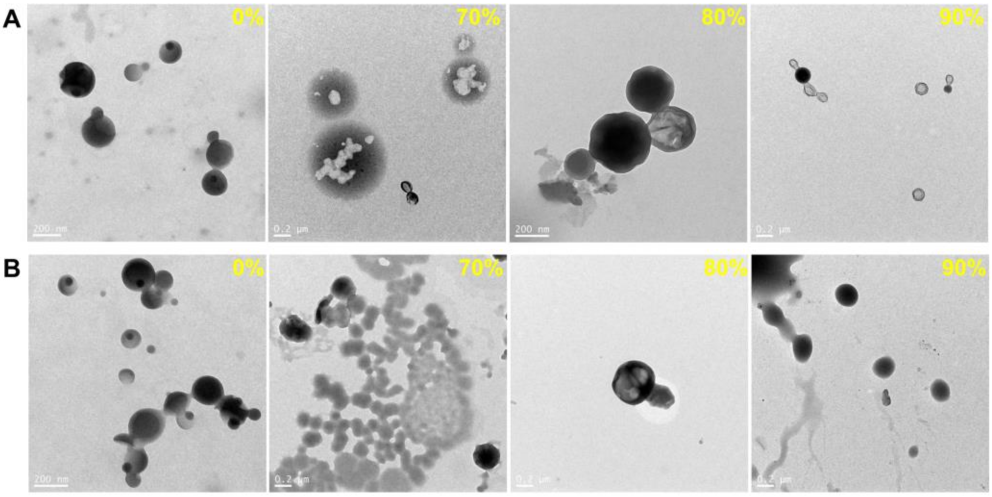

Figure S17. The TEM results of (A) DPAC-Py and (B) DPAC-$PyPF_6$ in the THF/water mixtures with different water fractions at room temperature, c = 10 μM. Scale bar: 200 nm.

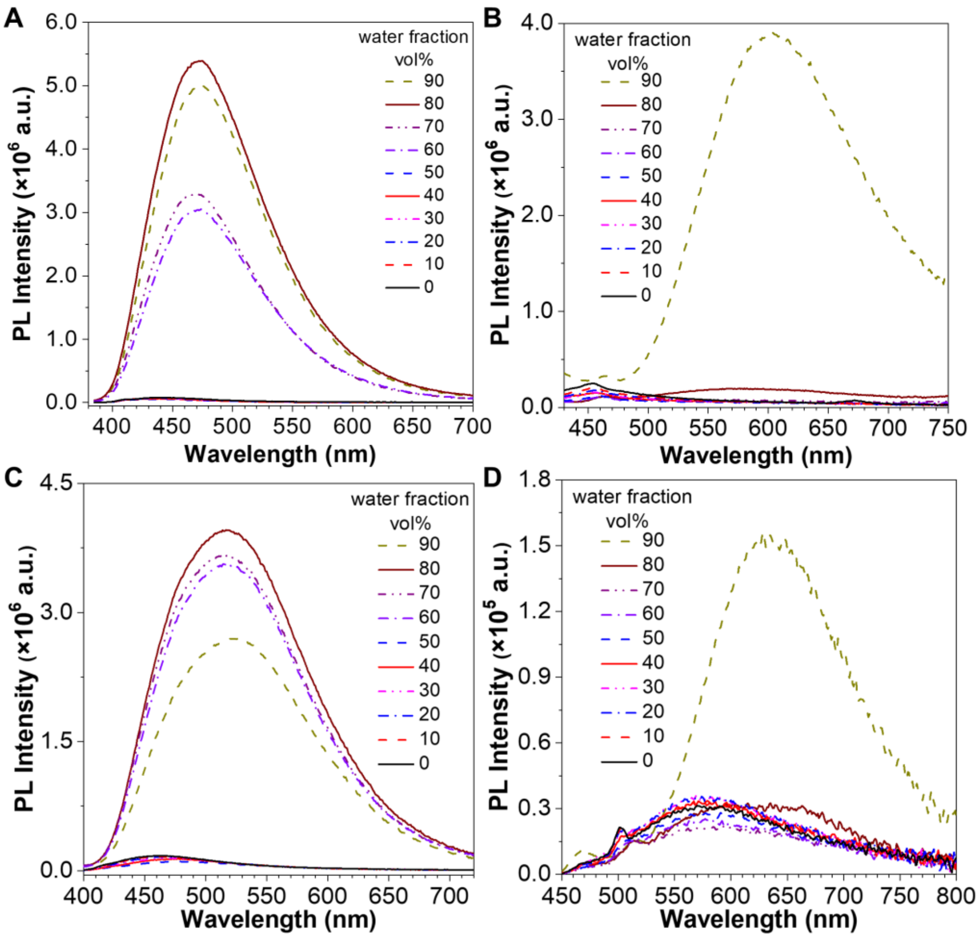

Figure S18. The fluorescence spectra of (A) DPAC-Py, (B) DPAC-$PyPF_6$, (C) DPAC-D-Py and (D) DPAC-D-$PyPF_6$ in the DMSO/water mixtures with different f_ws at room temperature, c = 10 μM, $\lambda_{ex,A}$ = 365 nm, $\lambda_{ex,B}$ = 401 nm, $\lambda_{ex,C}$ = 372 nm, $\lambda_{ex,D}$ = 438 nm.

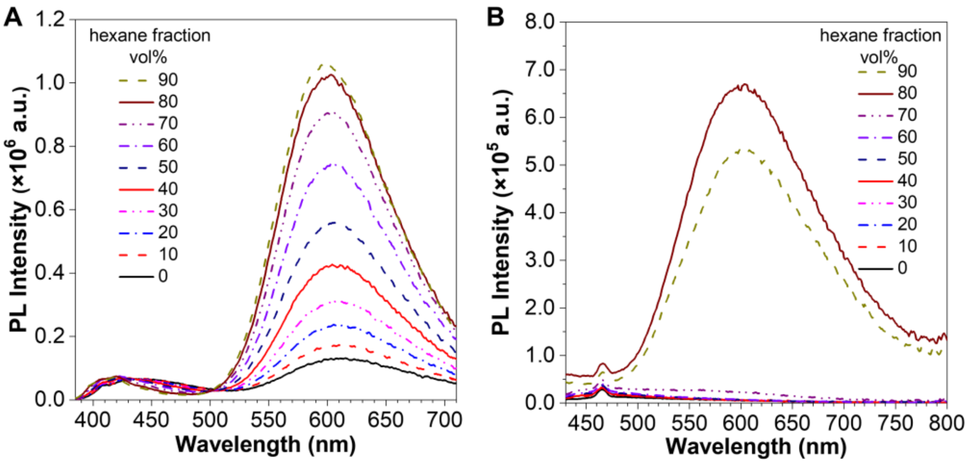

Figure S19. The FL spectra of (A) DPAC-Py and (B) DPAC-$PyPF_6$ in the THF/hexane mixtures with different hexane fractions (f_hs) at room temperature, c = 10 μM, $\lambda_{ex,A}$ = 365 nm, $\lambda_{ex,B}$ = 409 nm.

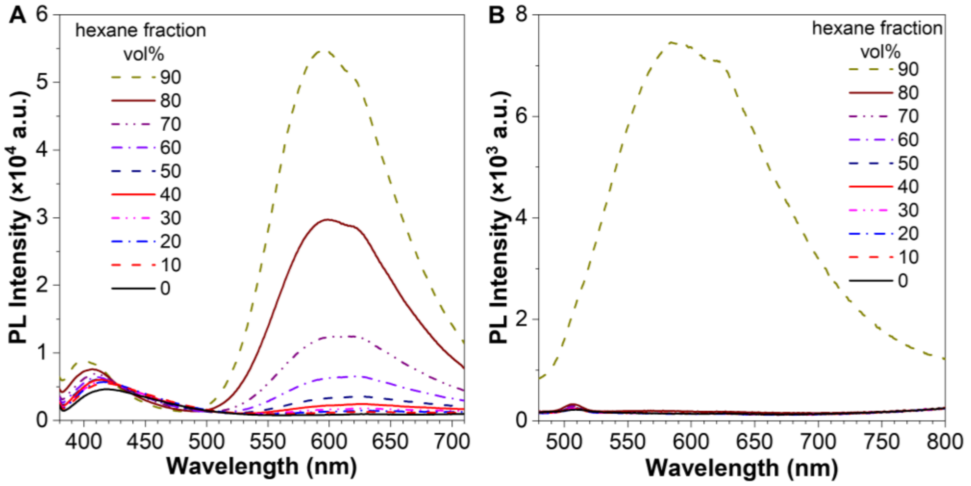

Figure S20. The FL spectra of (A) DPAC-Py and (B) DPAC-$PyPF_6$ in the DCM/hexane mixtures with different hexane fractions (f_hs) at room temperature, c = 10 μM, $\lambda_{ex,A}$ = 365 nm, $\lambda_{ex,B}$ = 440 nm.

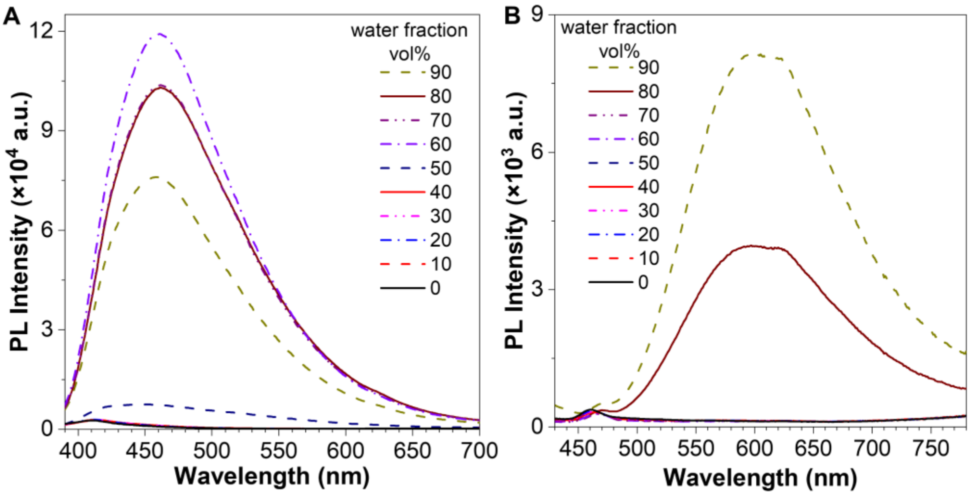

Figure S21. The FL spectra of (A) DPAC-Py and (B) DPAC-$PyPF_6$ in the MeOH/water mixtures with different water fractions (f_ws) at room temperature, c = 10 μM, $\lambda_{ex,A}$ = 365 nm, $\lambda_{ex,B}$ = 401 nm.

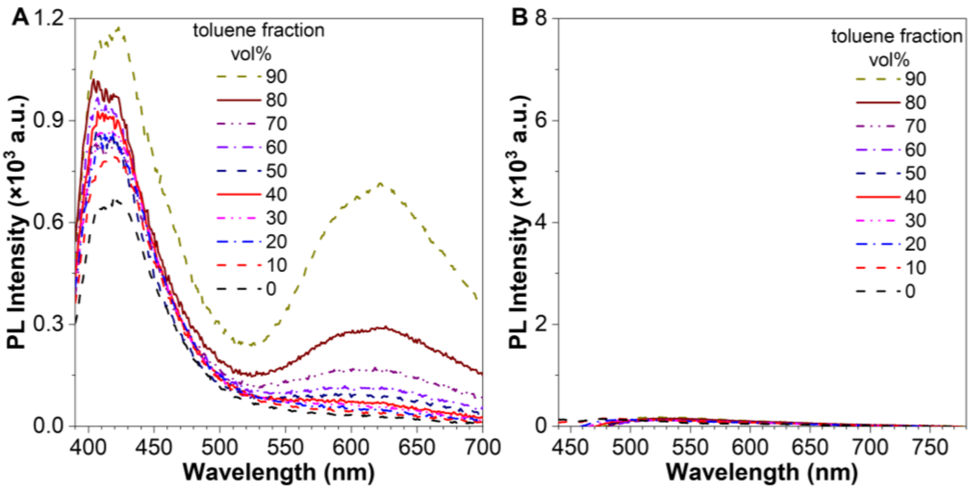

Figure S22. The FL spectra of (A) DPAC-Py and (B) DPAC-$PyPF_6$ in the MeOH/toluene mixtures with different toluene fractions (f_Ts) at room temperature, c = 10 μM, $\lambda_{ex,A}$ = 365 nm, $\lambda_{ex,B}$ = 401 nm.

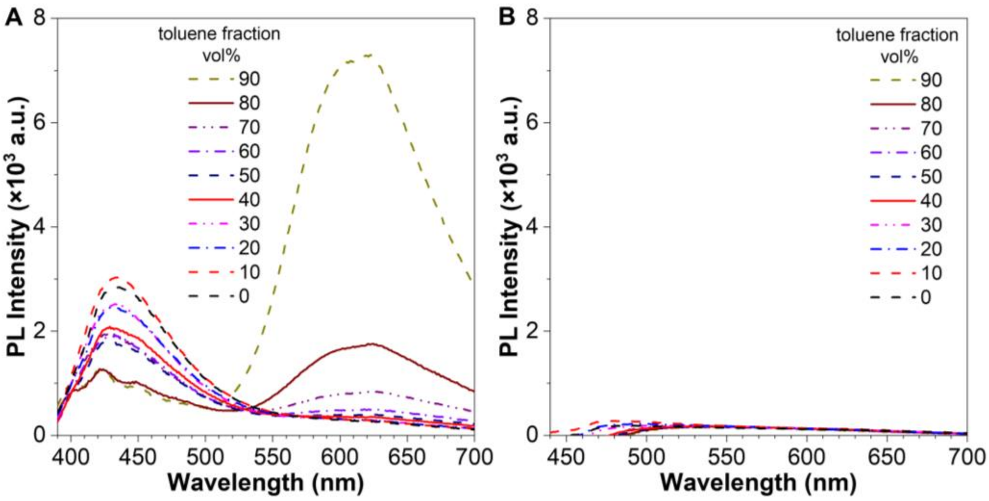

Figure S23. The FL spectra of (A) DPAC-Py and (B) DPAC-$PyPF_6$ in the DMSO/toluene mixtures with different toluene fractions (f_Ts) at room temperature, c = 10 μM, $\lambda_{ex,A}$ = 365 nm, $\lambda_{ex,B}$ = 401 nm.

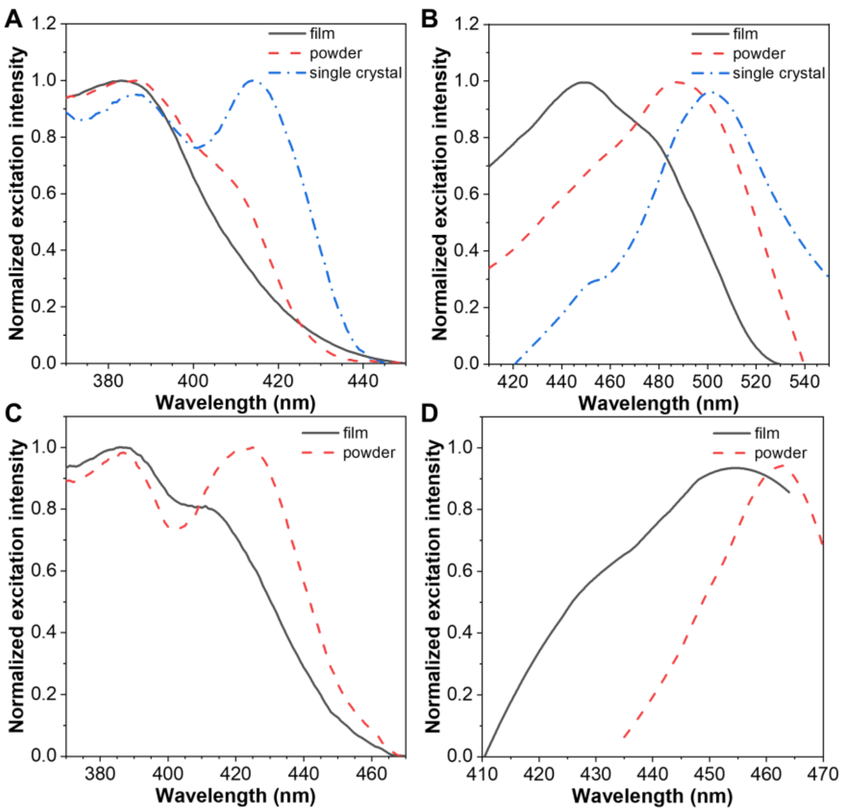

Figure S24. The normalized solid-state excitation spectra of (A) DPAC-Py, (B) DPAC-$PyPF_6$, (C) DPAC-D-Py and (D) DPAC-D-$PyPF_6$.

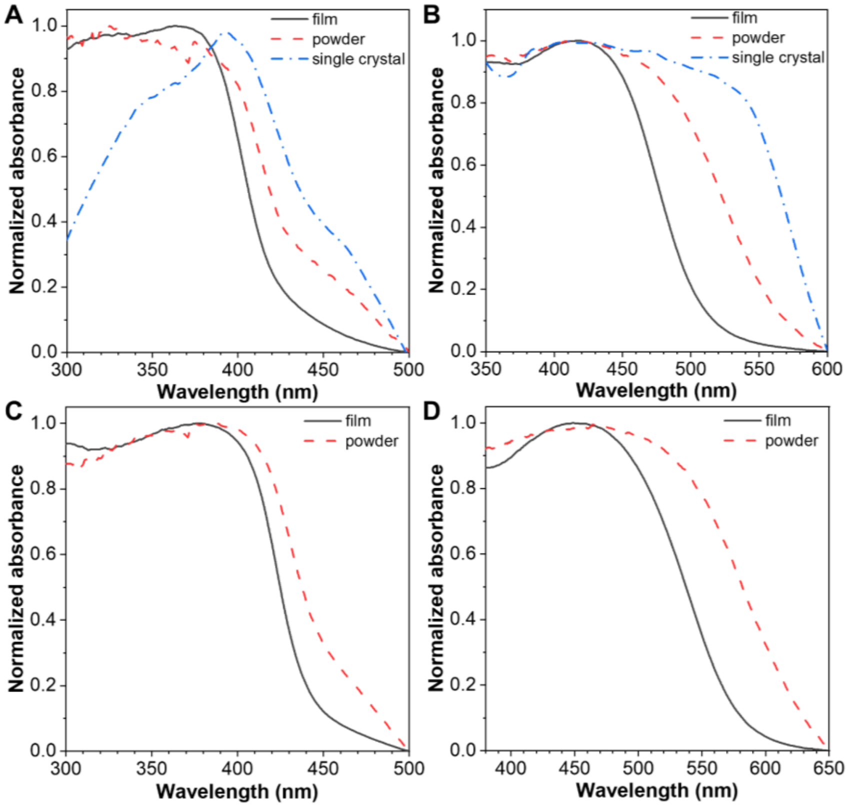

Figure S25. The normalized solid-state absorbance spectra of (A) DPAC-Py, (B) DPAC-$PyPF_6$, (C) DPAC-D-Py and (D) DPAC-D-$PyPF_6$.

Electrochemical Properties of the DPAC Derivatives

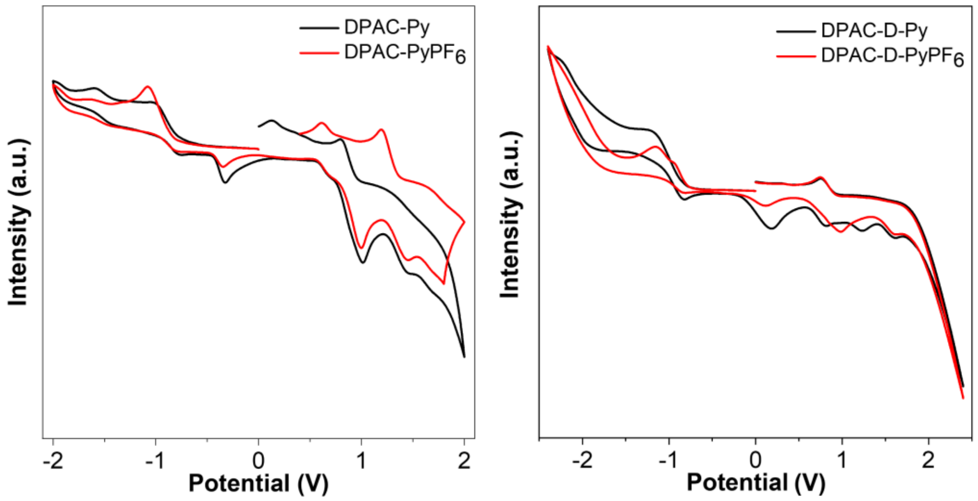

Figure S26. Cyclic voltammograms of DPAC-Py, DPAC-$PyPF_6$, DPAC-D-Py and DPAC-D-$PyPF_6$ in THF. Electrolyte solution: tetrabutylammonium hexafluorophosphate, scan rate: 0.1 V/s.

Theoretical Calculations of the DPAC Derivatives

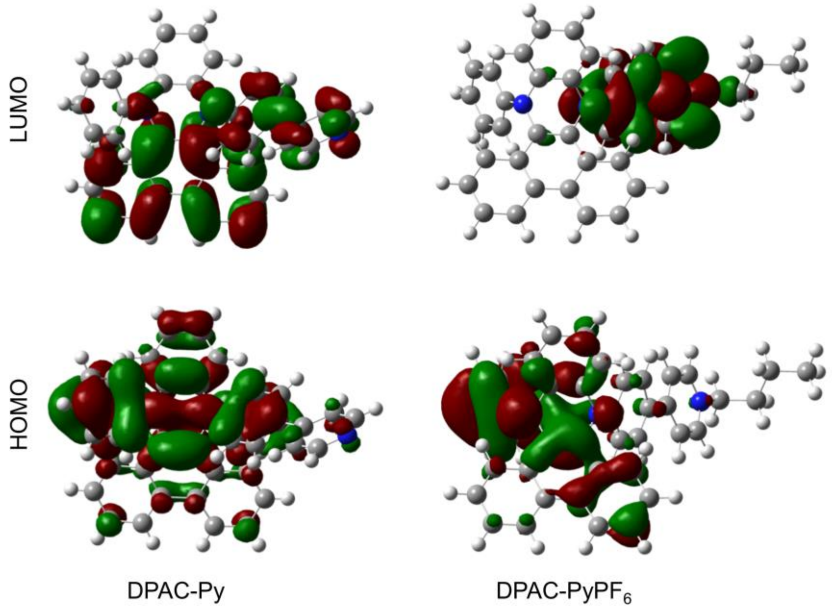

Figure S27. The HOMOs and LUMOs of DPAC-Py and DPAC-$PyPF_6$ calculated by DFT simulation based on the single-crystal structures.

Photosensitizing Properties of the DPAC Derivatives

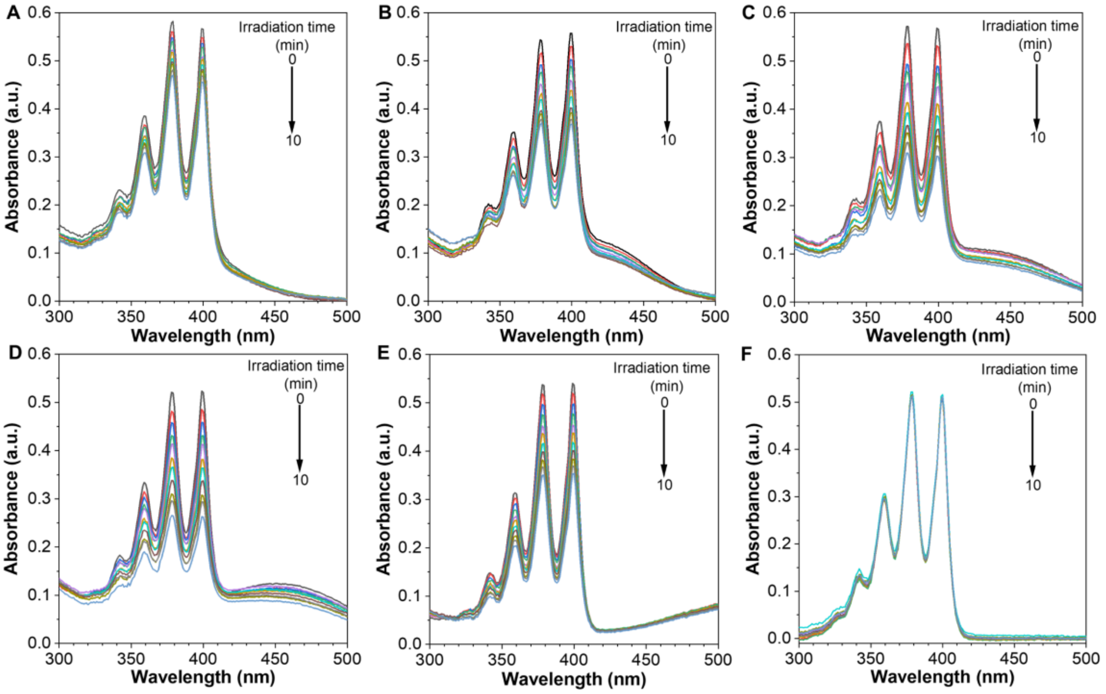

Figure S28. (A–E) Absorption spectra of (A) DPAC-Py, (B) DPAC-$PyPF_6$, (C) DPAC-D-Py, (D) DPAC-D-$PyPF_6$, (E) Rose Bengal in the presence of ABDA. (F) The absorption spectra of ABDA itself in aqueous solution (1 vol% DMSO) after being subjected to different time of white-light irradiation (10 mW/cm^2). [DPAC-Py] = [DPAC-$PyPF_6$] = [DPAC-D-Py] = [DPAC-D-$PyPF_6$] = 10 μM. [ABDA] = 50 μM.

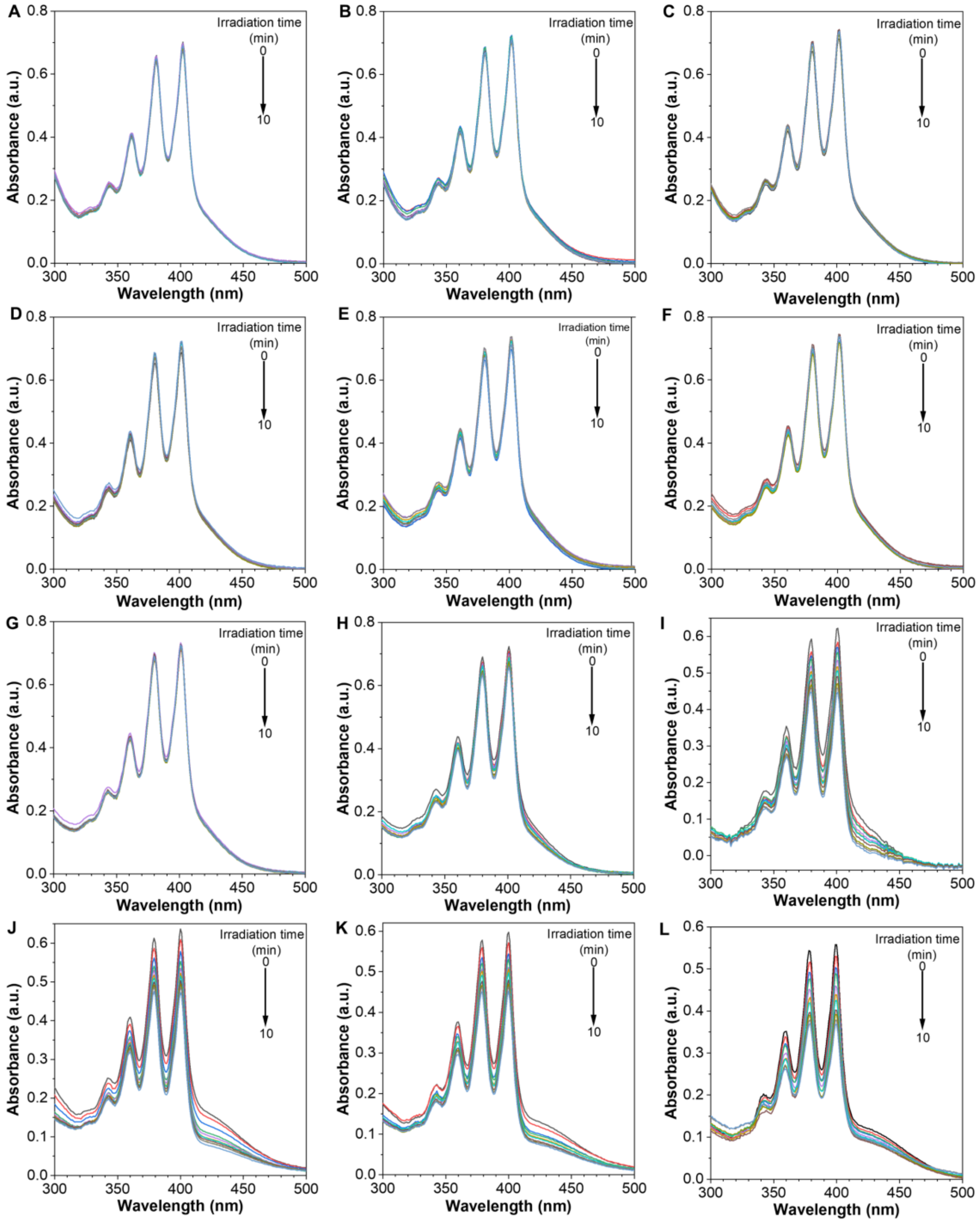

Figure S29. Absorption spectra of DPAC-$PyPF_6$ (10 μM) with ABDA (50 μM) in the mixtures of DMSO/water with different water fractions, (A) 0 vol%, (B) 10 vol%, (C) 20 vol%, (D) 30 vol%, (E) 40 vol%, (F) 50 vol%, (G) 60 vol%, (H) 70 vol%, (I) 80 vol%, (J) 90 vol%, (K) 95 vol%, (L) 99 vol% under white-light irradiation (10 mW/cm^2) for different time.

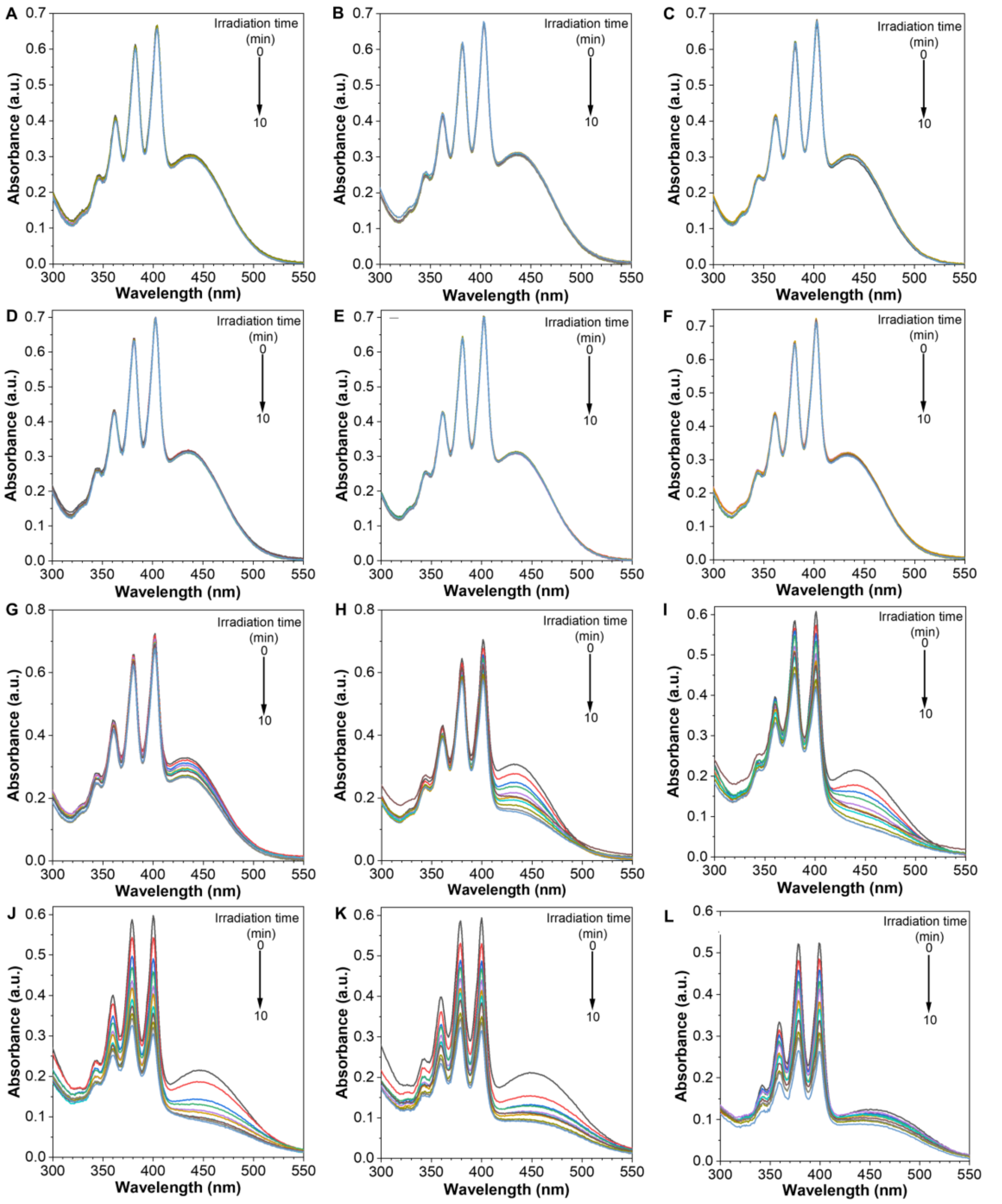

Figure S30. Absorption spectra of DPAC-D-PyPF$_6$ (10 μM) with ABDA (50 μM) in the mixtures of DMSO/water with different water fractions, (A) 0 vol%, (B) 10 vol%, (C) 20 vol%, (D) 30 vol%, (E) 40 vol%, (F) 50 vol%, (G) 60 vol%, (H) 70 vol%, (I) 80 vol%, (J) 90 vol%, (K) 95 vol%, (L) 99 vol% under white-light irradiation (10 mW/cm^2) for different time.

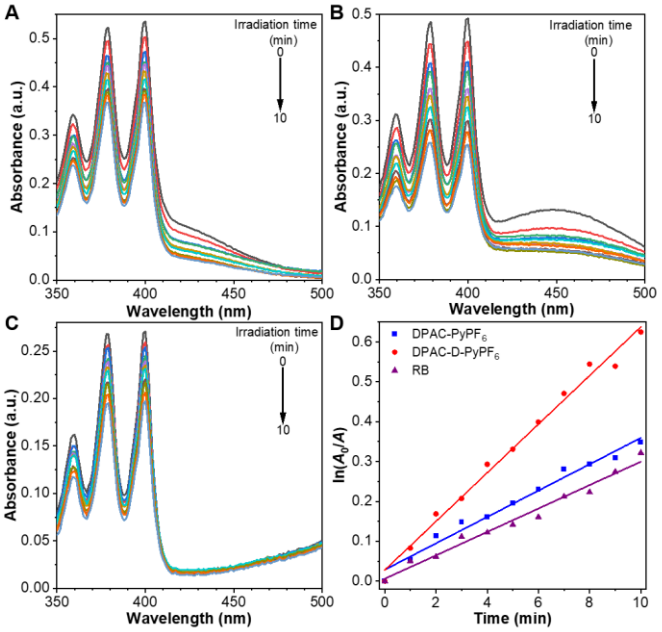

Figure S31. The UV-Vis spectra of ABDA in the presence of (A) DPAC-$PyPF_6$, (B) DPAC-D-$PyPF_6$ and (C) RB in aqueous solution (1 vol% DMSO) after being subjected to different time of white-light irradiation (10 mW/cm^2). (D) Time-dependent $^1O_2$ generation kinetics deduced from the decomposition rates of ABDA in the presence of different PSs under white-light irradiation (10 mW/cm^2) in aqueous solution (1 vol% DMSO). A_0 = absorption of ABDA at 378 nm without light irradiation. A = real-time absorbance of ABDA at 378 nm at different irradiation time. [ABDA] = 5×[PS]. To avoid the inner-filter effect, the absorption maxima of the PSs were adjusted to about 0.2 OD, and the corresponding concentrations were applied for the measurements.

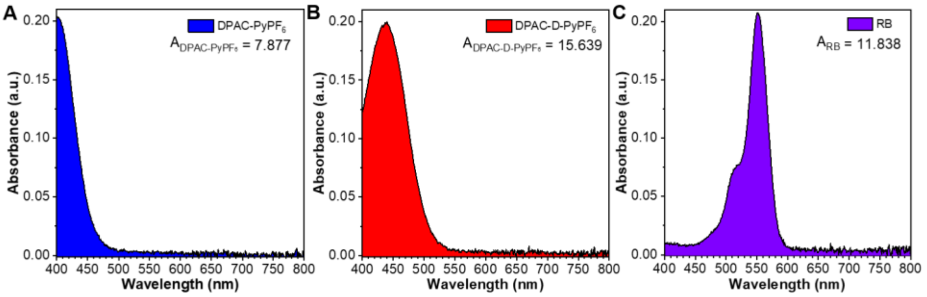

	Slope		Statistics	$^1O_2$ Quantum yield
	Value	Standard Error	Adj. R-Square	
**RB**	0.03308	0.00161	0.9768	0.75
**DPAC-$PyPF_6$**	0.06100	0.00209	0.9884	1.27
**DPAC-D-$PyPF_6$**	0.02938	0.00141	0.9773	1.18

Figure S32. The UV-Vis spectra of (A) DPAC-$PyPF_6$, (B) DPAC-D-$PyPF_6$ and (C) Rose Bengal in the range of 400–800 nm. The table shows the optical parameters of these photosensitizers calculated according to ln (A_0/A).

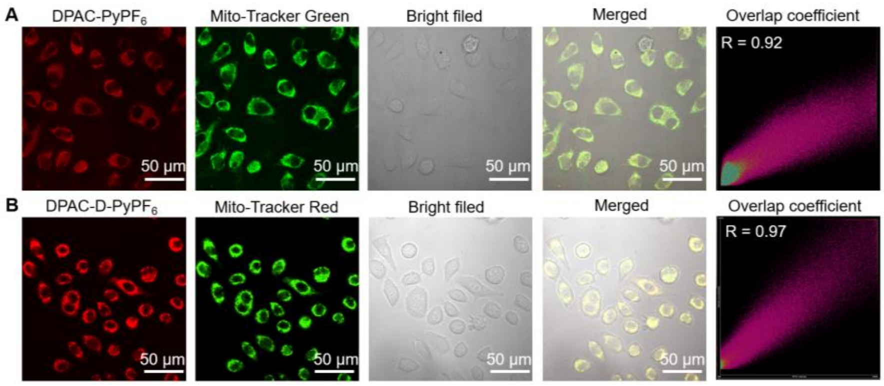

Figure S33. (A) Evaluation of the mitochondria-targeting ability of DPAC-PyPF$_6$ in HeLa cells. $\lambda_{ex, probe}$ = 404 nm, $\lambda_{em, probe}$ = 570–620 nm, $\lambda_{ex, Mito}$ = 488 nm, $\lambda_{em, Mito}$ = 500–549 nm. (B) Evaluation of the mitochondria-targeting ability of DPAC-D-PyPF$_6$ in HeLa cells. $\lambda_{ex, probe}$ = 404 nm, $\lambda_{em, probe}$ = 650–700 nm $\lambda_{ex, Mito}$ = 561 nm, $\lambda_{em, Mito}$ = 570–620 nm. Scale bar: 10 μm. [probe] = 5 μM, [Mito–Tracker] = 250 nM, Scale bar: 50 μm.

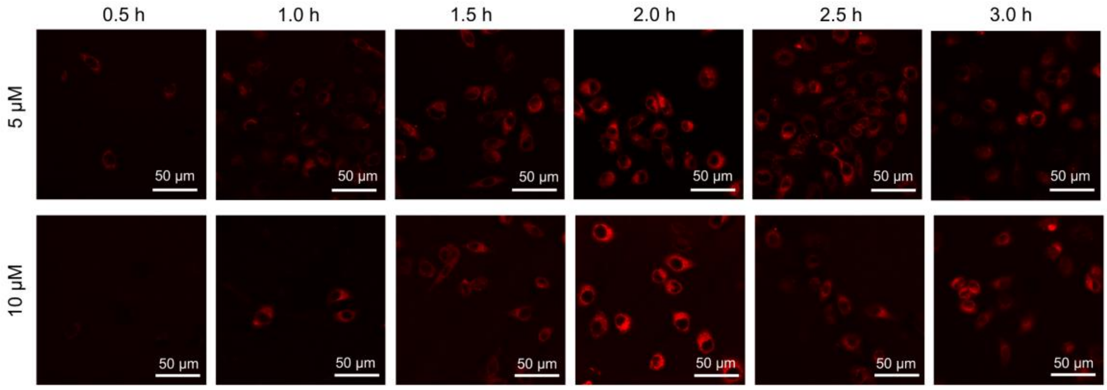

Figure S34. The CLSM of DPAC-PyPF$_6$ incubated with HeLa cells at 5 μM or 10 μM for different time. Scale bar: 50 μm.

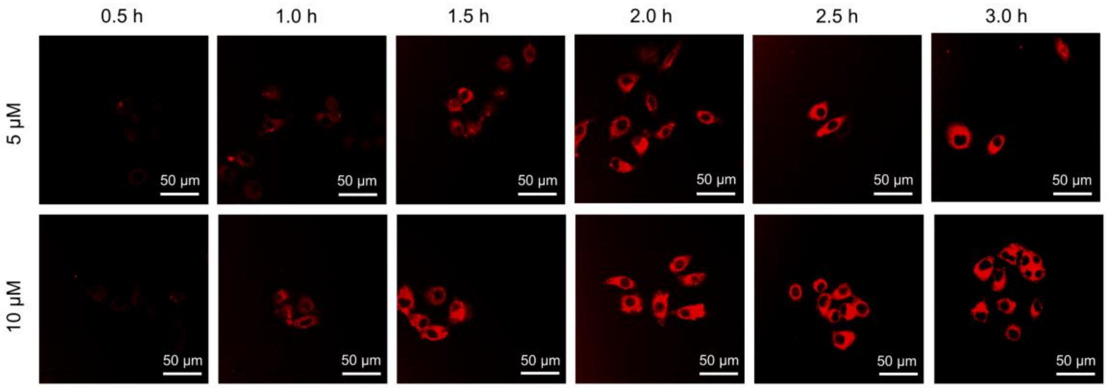

Figure S35. The CLSM of DPAC-D-PyPF$_6$ incubated with HeLa cells at 5 μM or 10 μM for different times. Scale bar: 50 μm.
